# Rif2 interaction with Rad50 counteracts Tel1 functions in checkpoint signalling and DNA tethering by releasing Tel1 from MRX binding

**DOI:** 10.1093/nar/gkad1246

**Published:** 2024-01-05

**Authors:** Paolo Pizzul, Erika Casari, Carlo Rinaldi, Marco Gnugnoli, Marco Mangiagalli, Renata Tisi, Maria Pia Longhese

**Affiliations:** Dipartimento di Biotecnologie e Bioscienze, Università degli Studi di Milano - Bicocca, 20126 Milano, Italy; Dipartimento di Biotecnologie e Bioscienze, Università degli Studi di Milano - Bicocca, 20126 Milano, Italy; Dipartimento di Biotecnologie e Bioscienze, Università degli Studi di Milano - Bicocca, 20126 Milano, Italy; Dipartimento di Biotecnologie e Bioscienze, Università degli Studi di Milano - Bicocca, 20126 Milano, Italy; Dipartimento di Biotecnologie e Bioscienze, Università degli Studi di Milano - Bicocca, 20126 Milano, Italy; Dipartimento di Biotecnologie e Bioscienze, Università degli Studi di Milano - Bicocca, 20126 Milano, Italy; Dipartimento di Biotecnologie e Bioscienze, Università degli Studi di Milano - Bicocca, 20126 Milano, Italy

## Abstract

The yeast Rif2 protein is known to inhibit Mre11 nuclease and the activation of Tel1 kinase through a short motif termed MIN, which binds the Rad50 subunit and simulates its ATPase activity *in vitro*. The mechanism by which Rif2 restrains Tel1 activation and the consequences of this inhibition at DNA double-strand breaks (DSBs) are poorly understood. In this study, we employed AlphaFold-Multimer modelling to pinpoint and validate the interaction surface between Rif2 MIN and Rad50. We also engineered the *rif2-S6E* mutation that enhances the inhibitory effect of Rif2 by increasing Rif2-Rad50 interaction. Unlike *rif2*Δ, the *rif2-S6E* mutation impairs hairpin cleavage. Furthermore, it diminishes Tel1 activation by inhibiting Tel1 binding to DSBs while leaving MRX association unchanged, indicating that Rif2 can directly inhibit Tel1 recruitment to DSBs. Additionally, Rif2^S6E^ reduces Tel1-MRX interaction and increases stimulation of ATPase by Rad50, indicating that Rif2 binding to Rad50 induces an ADP-bound MRX conformation that is not suitable for Tel1 binding. The decreased Tel1 recruitment to DSBs in *rif2-S6E* cells impairs DSB end-tethering and this bridging defect is suppressed by expressing a Tel1 mutant variant that increases Tel1 persistence at DSBs, suggesting a direct role for Tel1 in the bridging of DSB ends.

## Graphical Abstract

**Graphical Abstract:**
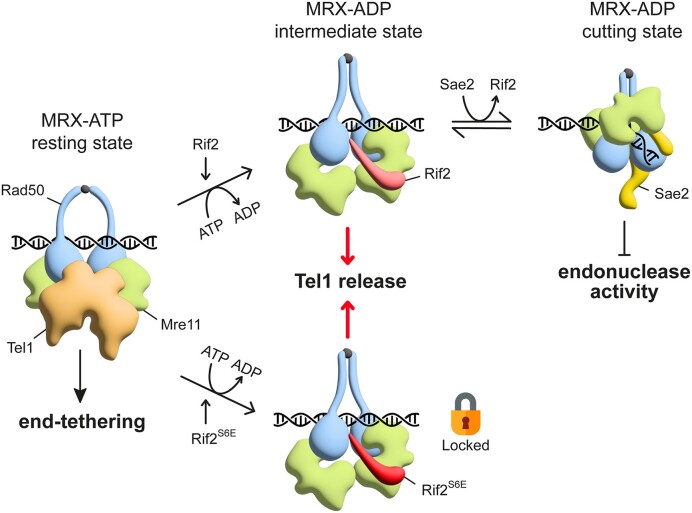

## Introduction

DNA double-strand breaks (DSBs) are cytotoxic lesions that can lead to cell death, gross chromosomal rearrangements, or loss of genetic information. Cells have evolved two major pathways to repair DNA DSBs: non-homologous end-joining (NHEJ) and homologous recombination (HR). In NHEJ, the DSBs are directly ligated with no or limited processing at their ends (1), while in HR the broken DNA ends are nucleolytically degraded to generate 3′ single-stranded DNA (ssDNA) overhangs that pair with intact homologous DNA templates (2). The generation of DNA DSBs also elicits a checkpoint response, whose key players are the apical protein kinases Tel1 and Mec1, as well as their mammalian orthologs ATM and ATR, respectively (3). Upon DSB recognition, Tel1 and Mec1 transduce the checkpoint signals to the downstream effector kinases Rad53 and Chk1 (CHK2 and CHK1 in mammals, respectively), whose activation requires the protein Rad9 (53BP1 in mammals).

The highly conserved Mre11-Rad50-Xrs2/NBS1 (MRX in *Saccharomyces cerevisiae* and MRN in mammals) complex acts as a main sensor of DNA DSBs (4,5). At the molecular level, the core Mre11-Rad50 subcomplex exists as a hetero-tetrameric assembly, in which two Mre11 subunits interact with two Rad50 nucleotide-binding domains to form a globular head from which two long Rad50 coiled coils protrude (6–10). MRX/MRN is required to maintain the DSB ends tethered to each other (11–14), an activity that has been attributed to the ability of Rad50 coiled coils to form intermolecular complexes that bridge the DNA ends (15–18). In addition, MRX/MRN is necessary to initiate resection of DNA DSBs that possess various secondary DNA structures and protein blocks at their ends (19–22). This resection activity depends on the Mre11 subunit, whose endonuclease activity nicks the 5′ strand near the DNA end, whereas its 3′-5′ exonuclease activity generates a short 3′-overhang of up to 300 nucleotides in length (20–24). The ssDNA tail is then elongated in the 5′-3′ direction by the long-range resection nuclease Dna2 or Exo1 (25–28). Finally, MRX/MRN is required for the recruitment and activation of the Tel1/ATM kinase (29–34). In *S. cerevisiae*, Tel1, once loaded at DSBs by MRX, stabilizes MRX association with DSBs through a positive feedback loop (35,36).

The two Rad50 subunits in the dimer constitute the binding sites for two ATP molecules. ATP binding to Rad50 induces a conformational rotation that increases the binding affinity of the two Rad50 subunits (37–41). In the ATP-bound state (resting state), the Rad50 dimer blocks the access of Mre11 to double-stranded DNA (dsDNA), thus preventing the MRX ability to incise the DNA ends (18,40–42). This MRX conformation has been proposed to be the active state for Tel1 activation, as *rad50* mutations that destabilize the ATP-bound state also impair Tel1 signalling activity (42–44). Upon ATP hydrolysis, the Rad50 nucleotide-binding domains dissociate and Mre11 moves to one side of Rad50. This conformational change makes DNA accessible to the Mre11 nuclease active sites, thus licensing the endonucleolytic DNA cleavage by Mre11 (cutting state) (45). Activation of Mre11 endonuclease activity within the context of the MRX complex requires the Sae2 protein (CtIP in humans) that, once phosphorylated by cyclin-dependent kinases, interacts with Rad50, and was proposed to stabilize the nucleolytically active Mre11-Rad50 conformation (46–48).

Recent data indicate that the activity of the MRX complex is negatively regulated by Rif2, a *S. cerevisiae* protein originated from duplication of the essential *ORC4* gene (49). Rif2 inhibits the Mre11 endonuclease activity *in vitro* (50,51), MRX-mediated resection of telomeric DNA ends (52–54), and MRX association with both DSBs and telomeres (35,55). These Rif2 inhibitory functions depend on a short amino acid motif, called MIN (MRN-Inhibitor), which is part of the BAT domain (Blocks Addition of Telomeres), previously identified as responsible for the negative regulation of telomere length (56). The MIN motif inhibits Mre11 endonuclease activity and interacts with Rad50 in the same region where Rad50 binds Sae2 (50,51,57).

**Figure 1.**
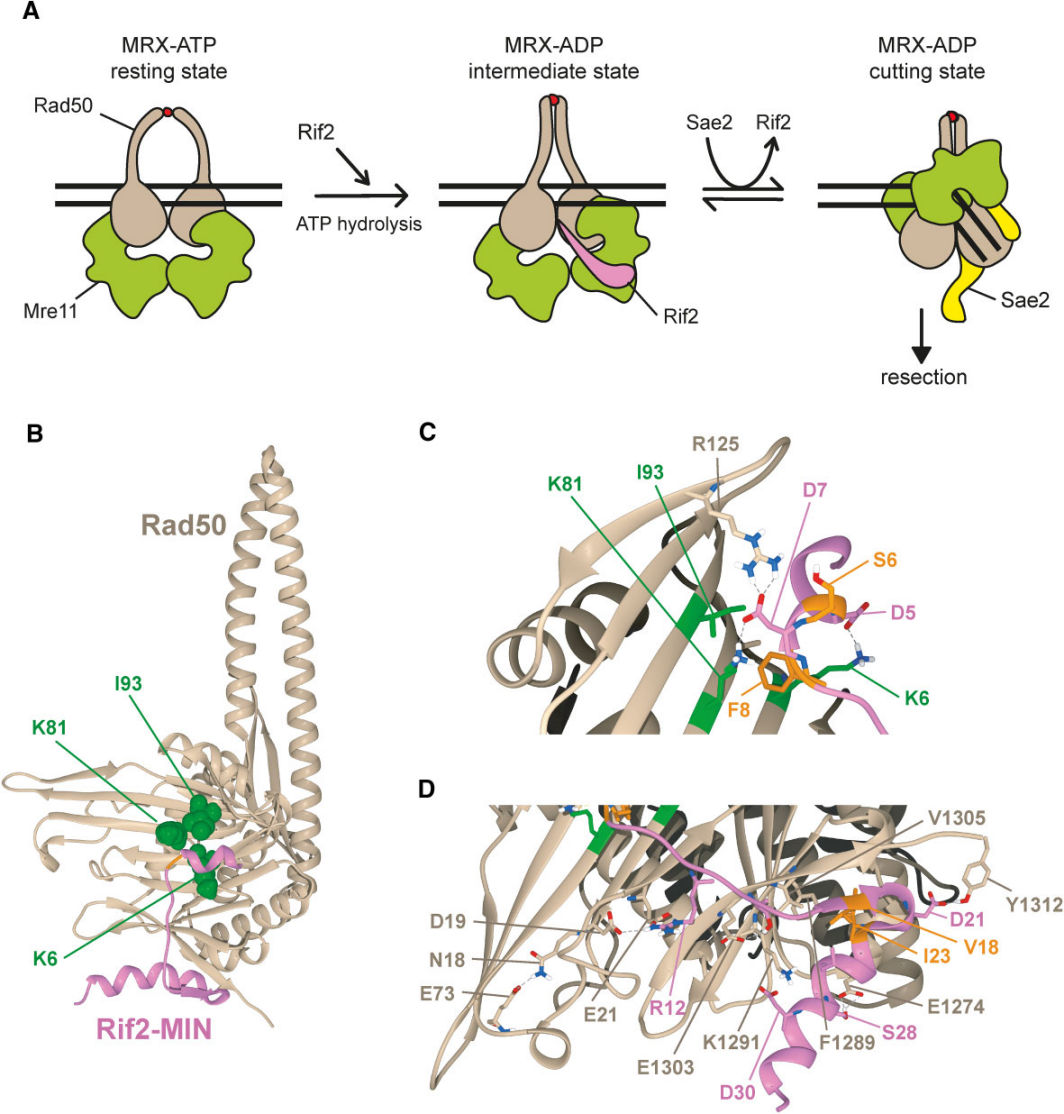
Model for regulation of MRX activity at DNA ends and AlphaFold-Multimer generated model of the Rad50 complex with Rif2 N-terminal 36 amino acids. (**A**) In the MRX ATP-bound state (resting state), Mre11 is inaccessible to dsDNA and therefore not competent to cleave it. Upon ATP hydrolysis, the two Rad50 coiled coils zip up and the Mre11 dimer rotates with respect to the Rad50 dimer globular domains. Rif2 binding to Rad50 stabilizes an Mre11-Rad50 ADP-bound state that is not competent for DNA cleavage. Sae2 antagonizes Rif2 binding to the Rad50-Mre11 interface and stabilizes an ADP-bound Mre11-Rad50 conformation (cutting state) that is proficient to cleave DNA. For simplicity, Xrs2 is not represented. The red dots indicate Zn^2+^ ions. (**B**) Cartoon representation of Rad50 (tan) and Rif2 N-terminal region (pink). The amino acids on the surface of Rad50 β-sheet identified as necessary for interaction with Rif2 MIN domain are represented as green spheres. (**C**) Detail of the interface between N-terminus of Rif2 and the β-sheets of Rad50. Rad50 residues previously known to be involved in Rif2-Rad50 interaction are in green. (**D**) Detail of the second interface identified. In both (C) and (D), residues in the interface are represented as sticks and Rif2 residues that were targeted for site-specific mutagenesis are in orange.

These data lead to a working model, as depicted in Figure 1A, in which MRX, in its ATP-bound conformation, can bind DNA but cannot cleave it. Following ATP hydrolysis by Rad50, the two Rad50 coiled coils zip up and Mre11 reaches the dsDNA by moving to the side of Rad50. Sae2 binding on the Rad50-Mre11 interface stabilizes this nucleolytically active ADP-bound MRX conformation, whereas Rif2 binding to Rad50 inhibits Mre11 nuclease activity by antagonizing the association of Sae2 with Rad50.

Interestingly, despite sharing no sequence homology with Rif2, the mammalian telomere-binding protein TRF2 carries an amino acid sequence, called iDDR (inhibitor of DNA Damage Response), that was recently found to interact with Rad50 similarly to MIN and to inhibit MRN-mediated resection of telomeric DNA ends (58).

In addition to limiting the Mre11 endonuclease activity, the Rif2 MIN motif counteracts Tel1-mediated telomere elongation by telomerase (50,56,57), MRX association at DNA ends (57), and MRX-dependent stimulation of Tel1 kinase *in vitro* (59). The mechanism through which Rif2 inhibits Tel1 activity at DNA ends is poorly understood, and various hypotheses can be conceived. Firstly, Rif2 might indirectly reduce Tel1 activation by diminishing the association of MRX with DSBs, which is necessary for recruiting Tel1 to DNA ends (29–34). Secondly, Rif2 might directly inhibit Tel1 activation by interfering with Tel1–MRX interaction. Thirdly, since Rif2 stimulates the Rad50 ATPase activity *in vitro* (35,59), this might lead to a conformational change that renders the MRX complex unable to stimulate Tel1 kinase or to bind Tel1 effectively.

To test these hypotheses, we initially used AlphaFold-Multimer to analyze the interaction interface between Rif2 MIN with Rad50. We then explored the effects of Rif2 binding to Rad50 by introducing mutations in Rif2 that enhance its interaction with Rad50. Our results show that the predicted Rad50-Rif2 interface involves hydrophobic residues conserved in the Rif2 yeast protein family but not in the mammalian TRF2 iDDR. Mutations of these residues reduce Rad50-Rif2 interaction and Rif2 functions at DNA ends. Furthermore, the substitution of S6 to E in Rif2 increases Rif2 affinity for Rad50 and enhances its inhibitory effect. In fact, unlike *rif2*Δ cells, *rif2-S6E* cells exhibit reduced hairpin cleavage. Furthermore, Rif2^S6E^ hampers Tel1 activation by reducing Tel1 association with DSBs without impairing MRX persistence at DSBs, indicating that Rif2 can directly reduce Tel1 recruitment to DNA ends. Rif2^S6E^ also diminishes Tel1-MRX interaction and stimulates ATPase by Rad50 more efficiently than wild-type Rif2, indicating that Rif2 binding to Rad50 counteracts Tel1 functions by inducing an MRX conformation unsuitable for Tel1 binding. In *rif2-S6E* cells, the decreased Tel1 association with DSBs leads to defects in maintaining the DSB ends close to each other, and this defect can be suppressed by enhancing Tel1 binding to DSBs. This finding, coupled with the observation that the role of Tel1 in supporting DSB end-bridging does not require its kinase activity, suggests a direct and structural role for Tel1 in DSB end-tethering.

## Materials and methods

### Yeast strains, plasmids, and growth conditions

*S. cerevisiae* is the experimental model used in this study. Strain genotypes are listed in Supplementary Table S1. Strains JKM139, used to detect DSB resection, and YMV45, used to detect DSB repair by SSA, were kindly provided by J. Haber (Brandeis University, Waltham, USA). Strain HS21, used to detect hairpin cleavage, was kindly provided by M.A. Resnick (NIH, Durham, USA). Strain YJK40.6, used to detect end-tethering, was kindly provided by D.P. Toczyski (University of California, San Francisco, USA). Strain YAB125 and plasmids pAB1214, pAB1243, pAB1245 and pAB2287, used for the two-hybrid assay, were kindly provided by A. Bianchi (University of Sussex, Brighton, UK). Plasmid pML801 was generated in this study. Gene disruptions and tag fusions were generated by one-step PCR and standard yeast transformation procedures. Primers used for disruptions and gene tagging are listed in Supplementary Table S2. Cells were grown in YEP medium (1% yeast extract, 2% bactopeptone) supplemented with 2% glucose (YEPD), 2% raffinose (YEPR), or 2% raffinose and 3% galactose (YEPRG). All experiments were performed at 26°C.

### Spot assays

Cells grown overnight were diluted to 1 × 10^7^ cells/ml. 10-fold serial dilutions were spotted on YEPD with or without DNA-damaging drugs. Plates were incubated for 2 or 3 days at 30°C.

### Southern blot analysis of telomere length

To determine the length of native telomeres, *Xho*I-digested genomic DNA was subjected to 0.8% agarose gel electrophoresis and hybridized with a ^32^P-labeled poly(GT) probe as previously described (60).

### Western blotting

Protein extracts for western blot analysis were prepared by trichloroacetic acid (TCA) precipitation. Frozen cell pellets were resuspended in 100 μl 20% TCA. After the addition of acid-washed glass beads, the samples were vortexed for 10 min. The beads were washed with 200 μl of 5% TCA twice, and the extract was collected in a new tube. The crude extract was precipitated by centrifugation at 850 × g for 10 min. TCA was discarded and samples were resuspended in 70 μl 2× Laemmli buffer (60 mM Tris pH 6.8, 2% SDS, 10% glycerol, 100 mM DTT, 0.2% bromophenol blue) and 30 μl 1 M Tris pH 8.0. Prior to loading, samples were boiled and centrifuged at 850 × g for 10 min. The supernatant containing the solubilized proteins was separated on 10% polyacrylamide gels. HA- or Myc-tagged proteins were detected by using anti-HA (12CA5) (1:2000) or anti-Myc (9E10) (1:1000) antibodies, respectively.

### Chromatin immunoprecipitation and qPCR

YEPR exponentially growing cell cultures of JKM139 derivative strains, carrying the HO cut site at the *MAT* locus, were transferred to YEPRG at time zero. Crosslinking was done with 1% formaldehyde for 5 min (Mre11), 10 min (Rad9), or 15 min (Tel1, Tel1^hy909^, Rif2, Rif2^S6E^). The reaction was stopped by adding 0.125 M glycine for 5 min. Immunoprecipitation was performed by incubating samples with Dynabeads Protein G (ThermoFisher Scientific) for 3 h at 4°C in the presence of 5 μg anti-HA (12CA5) antibodies or anti-Myc (9E10). Quantification of immunoprecipitated DNA was achieved by qPCR on a Bio-Rad CFX Connect^TM^ Real-Time System apparatus and Bio-Rad CFX Maestro 1.1 software. Triplicate samples in 20 μl reaction mixture containing 10 ng of template DNA, 300 nM for each primer, 2× SsoFast™ EvaGreen supermix (Bio-Rad #1725201) (2× reaction buffer with dNTPs, Sso7d-fusion polymerase, MgCl_2_, EvaGreen dye and stabilizers) were run in white 96-well PCR plates Multiplate™ (Bio-Rad MLL9651). The qPCR program was as follows: step 1, 98°C for 2 min; step 2, 90°C for 5 s; step 3, 60°C for 15 s; step 4, return to step 2 and repeat 40 times. At the end of the cycling program, a melting program (from 65 to 95°C with a 0.5°C increment every 5 s) was run to test the specificity of each qPCR. Data are expressed as fold enrichment at the HO-induced DSB over that at the non-cleaved *ARO1* locus, after normalization of the ChIP signals to the corresponding input for each time point. Fold enrichment was then normalized to the efficiency of DSB induction. Oligonucleotides used for qPCR analyses are listed in Supplementary Table S3.

### Coimmunoprecipitation

Total protein extracts were prepared as previously described (61). Briefly, cells were broken in 400 μl of buffer containing 50 mM HEPES pH 7.5, 140 mM NaCl, 1 mM EDTA pH 7.5, 10% glycerol, 1 mM sodium orthovanadate, 60 mM β‐glycerophosphate, 1 mM phenylmethylsulfonyl fluoride, and protease inhibitor cocktail (Roche Diagnostics). An equal volume of breaking buffer was added to clarified protein extracts and tubes were incubated for 2 h at 4°C with 50 μl of Protein G-Dynabeads and 5 μg anti-HA (12CA5) antibodies. The resins were then washed twice with 1 mL of breaking buffer. Bound proteins were visualized by western blotting with an anti-Myc (9E10) (1:1000) or an anti-HA (12CA5) (1:2000) antibody after electrophoresis on a 7.5% or 10% SDS-polyacrylamide gel.

### Yeast two-hybrid assay

Yeast two-hybrid assay was performed by co-transforming plasmids pAB1214 (GBD in pGBKT7), pAB1243 (GAD-*RIF2* in pGADT7), pAB1245 (GAD in pGADT7), pAB2287 (GBD-*RAD50-HEAD* in pGBKT7) and pML801 (GAD-*rif2-I23E* in pGADT7) in various combinations in YAB125 cells. Cells grown overnight in a selective medium were diluted 1 × 10^8^ cells/mL and 10-fold dilutions were spotted on synthetic complete (SC) medium lacking both tryptophan and leucine, and with or without histidine. Plates were incubated at 30°C for three (SC -leu -trp) or four (SC -leu -trp -his) days.

### DSB repair by SSA

DSB repair by SSA was detected in YMV45 derivative strains by Southern blot analysis using an *Asp*718-*Sal*I fragment containing part of the *LEU2* gene as a probe as previously described (62). Quantitative analysis of DSB repair by SSA was determined by calculating the ratio of band intensities for SSA to the total amount of SSA and DSB products for each time point. To normalize according to cut efficiency, the value of the uncut band was subtracted from the total amount of SSA and DSB products.

### DSB resection

YEPR exponentially growing cell cultures of JKM139 derivative strains, carrying the HO-cut site at the *MAT* locus, were transferred to YEPRG at time zero. *Ssp*I-digested genomic DNA was run on an alkaline agarose gel and visualized after hybridization with an RNA probe that anneals with the unresected strand on one side of the HO-induced DSB as previously described (63). This probe was obtained by *in vitro* transcription using Promega Riboprobe System-T7 and plasmid pML514 as a template. Plasmid pML514 was constructed by inserting in the pGEM7Zf vector a 900-bp fragment containing part of the *MAT* locus (coordinates 200870 to 201587 on chromosome III). Quantitative analysis of DSB resection was performed by calculating the ratio of band intensities for ssDNA and the total amount of DSB products. The resection efficiency was normalized with respect to the HO cleavage efficiency for each time point. Densitometric analysis of band intensities was performed using Scion Image Beta 4.0.2.

### Structural bioinformatics

Computational structural models for the complex between the 1–36 amino acid region of *S. cerevisiae* Rif2 and Rad50 monomer were built by AlphaFold-Multimer v3 on Colab Pro (https://colab.research.google.com/github/sokrypton/ColabFold/blob/main/AlphaFold2.ipynb) with pb100 template mode. The confidence of the models was assessed by the LDDT parameter and multimer metric, with the following final parameters for the top rank model: pLDDT = 85.8, pTM = 0.832, ipTM = 0.768. Visual inspection was performed to assess that the amino acids previously identified as directly involved in the interaction were part of the predicted interface, in detail F8 for Rif2 and K6, K81, and I93 for Rad50 (50,51,57). Structures were visualized with UCSF Chimera 1.17 (https://www.cgl.ucsf.edu/chimera/). Structural superposition was also achieved with UCSF Chimera.

### Protein purification and ATPase assay

Rad50 and Mre11 were expressed in yeast cells and purified as previously described (35). To assemble the Mre11-Rad50 complex, Rad50 and Mre11 were incubated together for 5 h on ice and separated from unassembled proteins in a Sephacryl S-400 gel filtration column. To express recombinant Rif2 and Rif2^S6E^, *RIF2* and *rif2-S6E* genes were chemically synthetized (GenScript, Piscataway, NJ, USA), cloned in frame with a C-terminal 6xHis-Tag into the pET21a vector and introduced in *Escherichia coli* BL21 (DE3) cells. To purify Rif2 and Rif2^S6E^, the corresponding proteins were produced in ZYM-5052 medium supplemented with ampicillin (100 mg/L), extracted and purified as previously described (35). Fractions containing the highest amount of protein were pooled and buffer-exchanged with 10 mM ammonium acetate pH 7.0 by gel filtration on PD-10 columns (GE Healthcare, Little Chalfont, UK). Samples were lyophilized in a freeze-dryer (Heto FD1.0, Gemini BV, Apeldoorn, the Netherlands) and stored at −20°C. Protein concentration was determined with the Bradford assay (Bio-Rad, Hercules, USA), using bovine serum albumin as a standard. The ATPase assay was performed as previously described (35). Briefly, wild-type Mre11-Rad50 (100 nM), Rif2 (2 μM) and Rif2^S6E^ (2 μM) were used in the presence of 100-bp dsDNA (200 nM) and 50 μm [α-^32^P]ATP. Radioactive ATP and ADP were separated by thin layer chromatography.

### Statistical analysis

Statistical analysis was performed using Microsoft Excel Professional 365 software. *P*-values were determined by using an unpaired two-tailed *t*-test. No statistical methods or criteria were used to estimate the size or to include or exclude samples.

## Results

### Analysis of the binding interface between the Rif2 MIN and Rad50

Rif2 inhibits MRX-dependent stimulation of Tel1 activity *in vitro* and Tel1 function at telomeres *in vivo* through a motif, termed MIN, that is situated at the Rif2 N-terminus (1–36 residues) (50,51,57). The inhibitory activity of Rif2 on Tel1 activation might encompass several potential mechanisms, including indirect regulation by reducing MRX association with DNA ends, direct interference with Tel1-MRX interaction, and/or modulation of Rad50 ATPase activity. As the Rif2 MIN motif interacts with Rad50 (50,51,57), to understand how Rif2 inhibits Tel1 activation, first we used the AlphaFold-Multimer predictor to generate models for the 1–36 amino acid MIN motif of Rif2 in complex with the Rad50 monomer from *S. cerevisiae*. As expected, the predictor found several structures of similar proteins to exploit as templates for Rad50 modelling, but no template is available for Rif2 N-terminal region (Supplementary Figure S1A). However, the structural prediction for Rif2 N-terminal was evaluated as a good and high-confidence prediction (Supplementary Figure S1B and C).

The top-ranking model is shown in Figure 1B. The amino acids on the Rad50 surface that were previously identified as required for interaction with the Rif2 MIN domain (57) are present in the interface predicted by AlphaFold-Multimer (Figure 1B). All the polar contacts between the two proteins are visualized in Figure 1C and D, and several of them involve amino acids previously shown to be conserved in the yeast Rif2 protein family. In detail, D5 and D7, which are highly conserved in both Rif2 and Orc4 homologs (50,57), are involved, respectively, in salt bridges with Rad50 K6 and K81 that were formerly identified as required for Rad50-Rif2 interaction (Figure 1C) (51,50,57). The residue F8 in Rif2 is also invariant in Rif2 and Orc4 families, and its substitution with A impairs Rif2-Rad50 interaction and Rif2 function in the regulation of telomere length (50,56,57). In the model, the Rad50 F8 is within 6 Å to Rad50 K81, which allows a cation-π interaction. Moreover, F8 is also less than 5 Å from I93, which is close enough to generate van der Waals interactions (Figure 1C).

The Rif2 R12 conserved residue interacts with D19 and E21 amino acids of Rad50 (Figure 1D). It is interesting to notice that D19 is localized in a tight turn near the N18 residue, previously reported as affected by N18S mutation that impinges on Rad50 affinity for Rif2 binding (51). The model would suggest that N18 is important for the structural properties of this turn, more than for the direct interaction with Rif2.

Interestingly, the α-helix composed of amino acids 20–33 in Rif2 is positioned below the globular domain of Rad50, representing a new binding surface for Rif2 on the Rad50 C-terminal lobe (Figure 1D). The interaction is mediated by amino acids in Rif2 N-terminal domain that are conserved in Rif2 protein family but not in TRF2 iDDR (58). In detail, V18 and I23 face a hydrophobic cluster involving F1289 and V1305 of Rad50. The D21 and S28 partially conserved residues and the highly conserved D30 residue in Rif2 make contact, respectively, with Rad50 Y1312, E1274, and K1291.

Since this second interface could be inaccessible to Rif2 when MRX complex is in resting conformation, we superimposed the Rif2-Rad50 subcomplex to the model of the ATP-bound heterotetrameric state of Mre11-Rad50 that we previously described (44). The N-terminal of Rif2 occupies a space that is empty in the Mre11-Rad50 tetramer and should be accessible when the Mre11-Rad50 complex is in resting conformation, as required for Rif2 to stimulate Rad50 ATPase activity (Supplementary Figure S1D).

### Validation of the Rif2-Rad50 binding interface through the identification of MIN mutations that decrease or increase Rif2-Rad50 interaction

To further validate the predicted Rif2-Rad50 interacting region, we replaced the highly conserved Rif2 residues V18 and I23 with E to introduce a negative charge, which was expected to disrupt the hydrophobic cluster that forms the core of the second interface. Furthermore, we engineered mutations that could enhance Rif2 inhibitory functions by further stabilizing the interaction of Rif2 with Rad50. To increase Rif2-Rad50 affinity, we considered mutations in the first interface, in particular, the replacement of F8 with E. In fact, as F8 was predicted to interact with K81, its mutation to E might substitute the cation-π interaction with a salt bridge interaction. Besides, we decided to mutagenize the S6 residue to E to allow its interaction with the nearby Rad50 R125 residue (Figure 1C).

**Figure 2.**
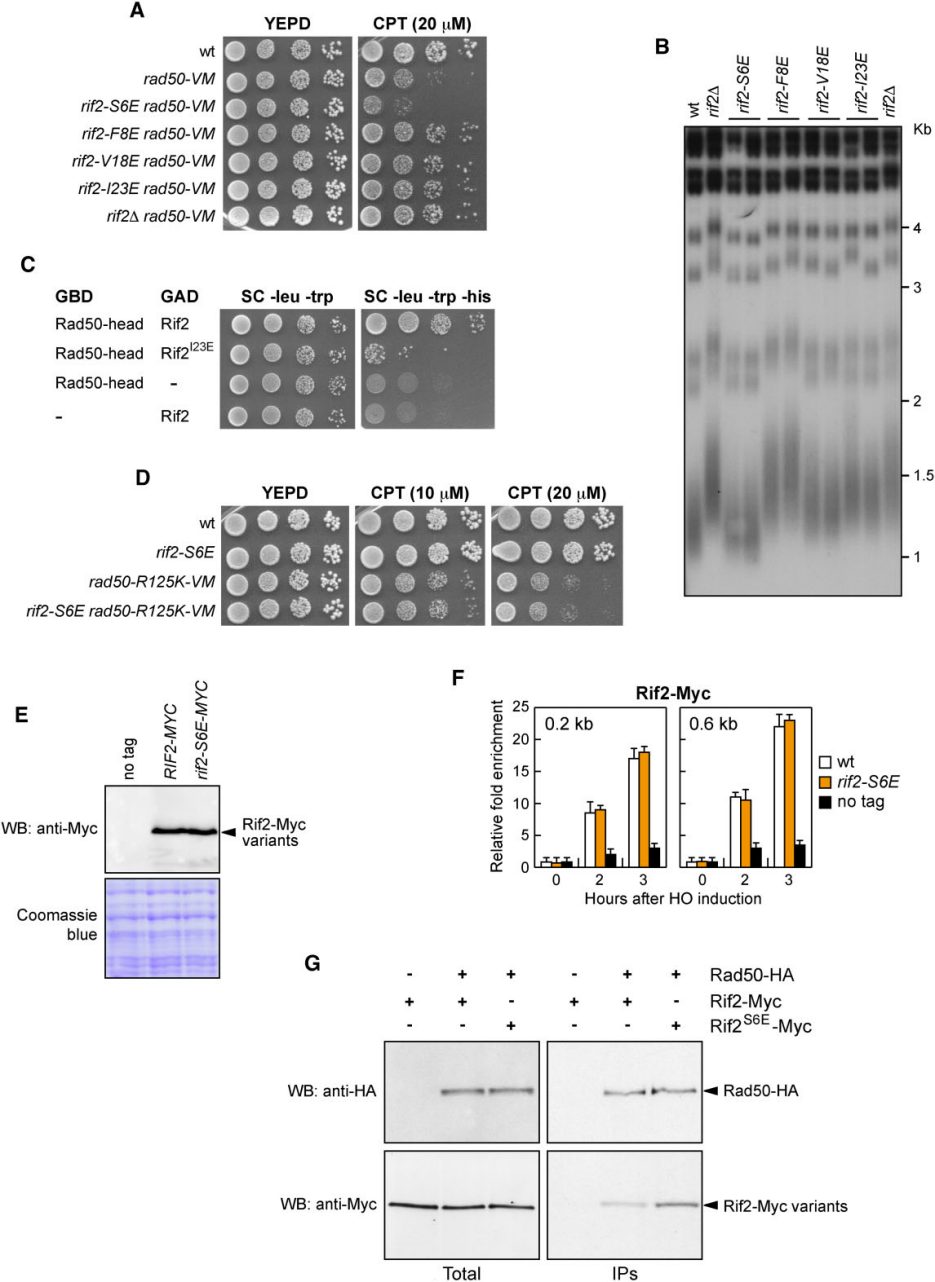
The *rif2-S6E* allele exacerbates the DNA damage sensitivity of *rad50-VM* cells and increases Rif2 interaction with Rad50. (**A**) Exponentially growing cultures were serially diluted (1:10) and each dilution was spotted out onto YEPD plates with or without camptothecin (CPT). (**B**) Telomere length. *Xho*I-cut genomic DNA from exponentially growing cells was subjected to Southern blot analysis using a poly(GT) telomere-specific probe. (**C**) Two-hybrid assay. Cells containing the indicated proteins fused to either GBD (Gal4 binding domain) or GAD (Gal4 activation domain) were spotted on synthetic complete (SC) medium lacking the indicated amino acids. (**D**) Exponentially growing cultures were serially diluted (1:10) and each dilution was spotted out onto YEPD plates with or without CPT. (**E**) Western blot with anti-Myc antibodies of extracts used for the ChIP analysis shown in (F). The same amount of protein extracts was separated on SDS-PAGE and stained with Coomassie blue as loading control. (**F**) Rif2-Myc ChIP and qPCR at the indicated distances from the HO-cut site. Data are expressed as fold enrichment at the HO-cut site over that at a non-cleavable locus (*ARO1*), after normalization to the corresponding input for each time point. Fold enrichment was normalized to cut efficiency. Plotted values are the mean value of three independent experiments with error bars denoting standard deviation (s.d.). (**G**) Coimmunoprecipitation. Protein extracts from exponentially growing cells were analyzed by western blotting with anti-HA and anti-Myc antibodies either directly (Total) or after immunoprecipitation (IPs) of Rad50-HA with an anti-HA antibody.

The deletion of *RIF2* has been reported to partially restore the DNA damage resistance of cells carrying the *rad50-V1269M* allele (referred to as *rad50-VM*), which we previously identified by searching for mutations that sensitize *tel1*Δ cells to the type I topoisomerase inhibitor camptothecin (CPT) (35). The DNA damage sensitivity of *rad50-VM* cells was shown to be due to a decreased association of MR^VM^X with DNA DSBs, and the lack of Rif2 restored DNA damage resistance of *rad50-VM* cells by increasing MR^VM^X persistence at DSBs (35). Hence, to determine whether the above *rif2-V18E*, *rif2-I23E*, *rif2-F8E*,and *rif2-S6E* alleles abolish or enhance Rif2 function, we combined them with the *rad50-VM* allele. Our rationale was that the mutations resulting in Rif2 loss of function are expected to alleviate the DNA damage sensitivity of *rad50-VM* cells, whereas those inducing Rif2 hyperactivation should exacerbate it. In addition, as *RIF2* deletion results in overelongation of telomeres (64–66), telomere length was also assessed. Similar to *rif2*Δ, the *rif2-V18E* and *rif2-I23E* alleles partially restored resistance to CPT of *rad50-VM* cells (Figure 2A) and caused telomere overelongation (Figure 2B), indicating that they impair Rif2 function. Given that I23E is the mutation that causes the most severe overelongation of telomeres, we investigated whether this mutation impairs Rif2 function by reducing Rad50-Rif2 interaction. Utilizing a two-hybrid approach in cells that express the Rad50 head domain along with either wild-type Rif2 or Rif2^I23E^ (50), we found that *rif2-I23E* reduced the ability of Rif2 to interact with Rad50 (Figure 2C), thereby substantiating the critical role of this region in mediating the Rad50-Rif2 interaction.

Unfortunately, the *rif2-F8E* mutation, which was designed to increase Rif2 affinity for Rad50 and therefore the Rif2 inhibitory function, behaved similarly to *rif2-V18E* and *rif2-I23E* (Figure 2A and B), likely due to the impossibility of creating the right geometry for interaction with K81 or to the inadequate interaction with the hydrophobic pocket on the Rad50 surface (nearby I93) (Figure 1C).

Interestingly, the *rif2-S6E* allele, which was also designed to enhance Rif2-Rad50 interaction, did not lead to an increase in telomere length (Figure 2B) and exacerbated the DNA damage sensitivity of *rad50-VM* cells (Figure 2A), raising the possibility that it could enhance Rif2 functions. In the predicted Rad50-Rif2 binding region, S6 is nearby the Rad50 R125 residue (Figure 1C), which interacts with Rif2 D7. The substitution of S6 with E introduces a negative charge that allows the formation of a bond between Rif2 E6 and Rad50 R125, in addition to the already existing interaction of R125 with Rif2 D7. If the effect of the S6E substitution depends on a stronger interaction of Rif2 with Rad50 R125, then replacing R125 with K should only permit alternative interactions with D7 or with E6, but not with both, and therefore is expected to restore a wild-type Rif2-Rad50 affinity and to suppress the ability of *rif2-S6E* to exacerbate the *rad50-VM* DNA damage sensitivity. As shown in Figure 2D, *rif2-S6E rad50-R125K-VM* were as sensitive as *rad50-R125K-VM* cells, indicating that the *rif2-S6E* allele was unable to increase the CPT sensitivity of cells expressing a *rad50-VM* allele that also carries the R125K amino acid substitution. This finding demonstrates that the effect of the *rif2-S6E* mutation depends on a direct interaction between Rad50 and Rif2.

The *rif2-S6E* mutation increases neither Rif2 protein level nor Rif2 association with DSBs. In fact, similar amounts of Rif2 and Rif2^S6E^ were detected in protein extracts (Figure 2E). Furthermore, by using JKM139 derivative strains, where a single irreparable DSB at the *MAT* locus can be generated by expressing the site-specific HO endonuclease from a galactose-inducible promoter (67), similar amount of Rif2 and Rif2^S6E^ bound to DNA sequences close to the HO endonuclease cut site was found by chromatin immunoprecipitation (ChIP) and quantitative PCR (qPCR) (Figure 2F).

As expected from the predicted Rad50-Rif2 interaction interface, the *rif2-S6E* mutation indeed increases the interaction between Rif2 and Rad50. In fact, when we created yeast strains expressing epitope-tagged versions of Rad50, Rif2, and Rif2^S6E^ from their native genomic loci and Rad50-hemagglutinin (HA) was immunoprecipitated with an anti-HA antibody, an increase of Rif2^S6E^-Myc was detected in HA-tagged Rad50 immunoprecipitates compared with wild-type Rif2-Myc (Figure 2G).

Taken together, these results, supported by previous mutational analyses examining binding capability, provide experimental support for the accuracy of the predicted Rif2-Rad50 interaction region.

### Rif2^S6E^ reduces hairpin cleavage but not resection of an HO-induced DSB

Rif2 is less effective in inhibiting MRX-mediated resection and Tel1 activation at DSBs than at telomeres, where Rif2, in conjunction with Rif1 and Rap1 proteins, forms a higher-order architecture that provides a protective cap and limits telomerase access (68). To understand why Rif2 inhibition of MRX and Tel1 is kept low at DSBs compared to telomeric ends, we explored the consequences of expressing the Rif2^S6E^ variant that strengthens Rif2 interaction with Rad50 and potentially its inhibitory function.

**Figure 3.**
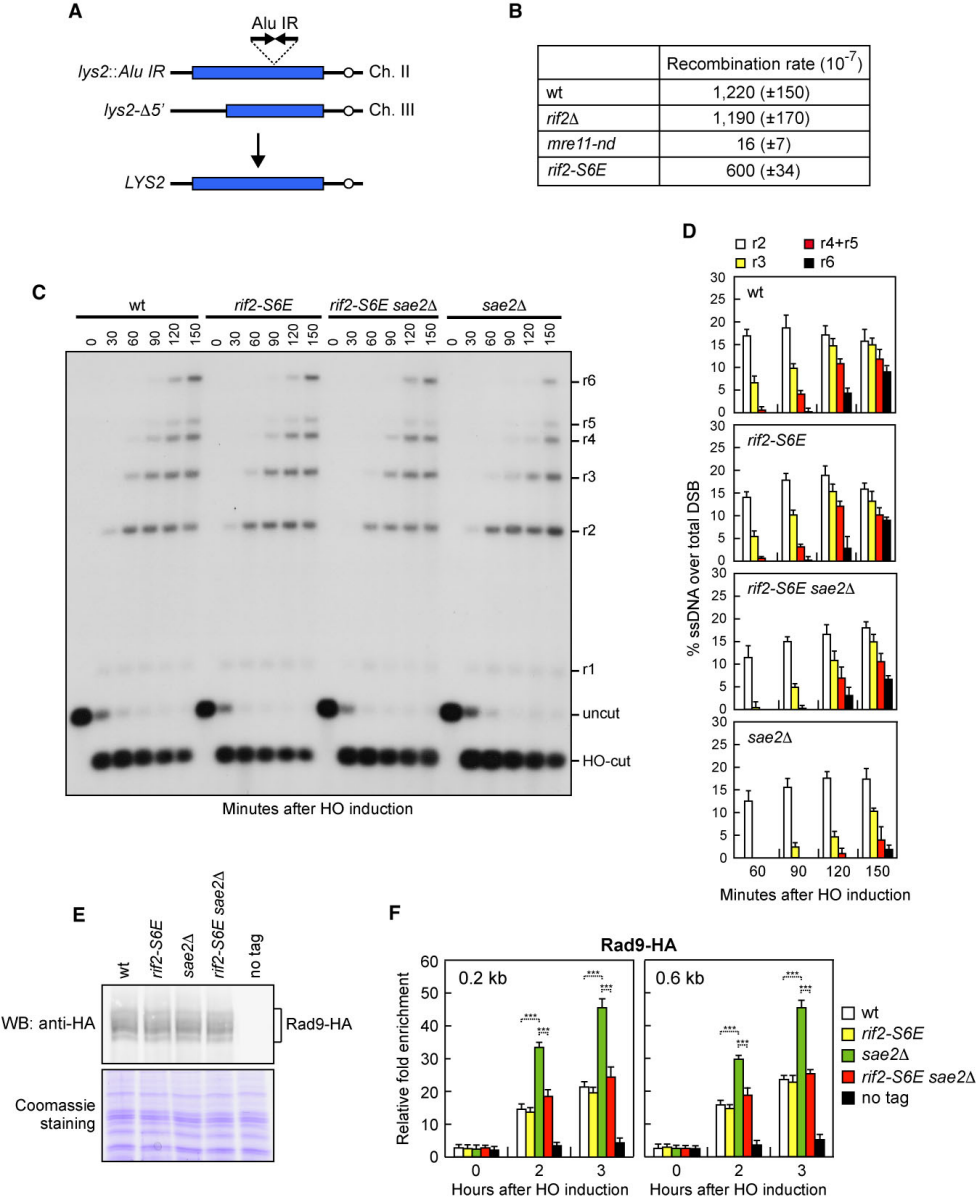
Hairpin resolution and resection of an HO-induced DSB in *rif2-S6E* cells. (**A**) Schematic representation of the *lys2::Alu IR* and *lys2-*Δ*5’* ectopic recombination system. (**B**) Recombination frequency of strains with the *lys2::Alu IR* and *lys2-*Δ*5’* ectopic recombination system. The rate of Lys^+^ recombinants was derived from the median recombination frequency. The reported values are the mean values of three independent experiments with s.d. indicated in brackets. (**C**) DSB resection. JKM139 derivative strains were transferred to YEPRG at time zero. *Ssp*I-digested genomic DNA was hybridized with a single-stranded *MAT* probe that anneals with the unresected strand. 5′-3′ resection produces *Ssp*I fragments (r1 through r6) detected by the probe. (**D**) Densitometric analysis of the resection products. The mean values of three independent experiments as in (C) are represented with error bars denoting s.d. (**E**) Western blot with an anti-HA antibody of extracts used for the ChIP analysis shown in (F). (**F**) Rad9-HA ChIP and qPCR at the indicated distance from the HO-cut site as described in Figure 2F. Plotted values are the mean value of three independent experiments with error bars denoting s.d. ***p< 0.005 (Student's *t*-test).

We and others have shown that Rif2 inhibits the Sae2-stimulated endonuclease activity of the Mre11-Rad50 subcomplex (50,51). The Mre11 endonuclease activity is essential for the processing of DSBs with non-canonical structures such as DNA hairpins or protein adducts (19–22), whereas it is dispensable at clean DSBs such as those generated upon induction of the HO endonuclease (69). To assess whether the *rif2-S6E* mutation increases Rif2 inhibition of Mre11 endonuclease activity, we analyzed the effect of this mutation on MRX-mediated hairpin resolution. To this purpose, we used a genetic assay in which inverted Alu elements inserted in the *LYS2* gene on chromosome II induce a hairpin-capped DSB that is cleaved by MRX-Sae2 and subsequently repaired by HR using a truncated *lys2* gene (*lys2-Δ5*′) located on chromosome III to generate Lys^+^ recombinants (Figure 3A) (19). As expected, the inverted Alu elements stimulate ectopic recombination and this stimulation depends on the Mre11 nuclease, as nuclease defective *mre11-H125N* (*mre11-nd*) cells strongly decreased the rate of Lys^+^ recombinants compared to wild-type cells (Figure 3B). The generation of Lys^+^ recombinants remained unaltered in *rif2*Δ cells, possibly because the low amount of Rif2 bound at DSBs compared to Sae2 is not enough to limit Sae2-mediated stimulation of Mre11 endonuclease. By contrast, it was decreased in *rif2-S6E* cells relative to wild-type cells (Figure 3B), suggesting that this allele increases Rif2 inhibition of MRX-mediated hairpin cleavage.

Consistent with previous findings that the nuclease activity of Mre11 is dispensable for the processing of clean DSBs such as those generated upon induction of the HO endonuclease (69), we anticipated that *rif2-S6E* cells were capable to resect an HO-induced DSB at the *MAT* locus (Figure 3C and D). To measure DSB resection, we used JKM139 derivative strains, where a single DSB at the *MAT* locus can be generated by expressing the HO endonuclease upon galactose addition (67). Resection of DNA regions flanking the HO-induced DSB renders the DNA sequence resistant to cleavage by restriction enzymes, resulting in the appearance of slower migrating bands (r1–r6) that can be detected after hybridization with a probe that anneals to the unresected strand on one side of the DSB. The appearance of the ssDNA intermediates at the HO-induced DSB occurred with similar kinetics in both wild-type and *rif2-S6E* cells (Figure 3C and D), indicating that the *rif2-S6E* mutation does not affect resection of the HO-induced DSB.

It has been shown that the lack of Sae2 impairs DSB resection because it enhances MRX/Tel1 signalling activity that leads to an increased Rad9 association with DSBs that, in turn, inhibits Exo1 and Dna2 nucleases (70–72). *rif2-S6E* cells possess wild-type levels of Rad9 both in protein extracts (Figure 3E) and bound at the HO-induced DSB (Figure 3F). Consistent with the suppression of *sae2*Δ resection defect, *rif2-S6E sae2*Δ cells showed a decreased Rad9 association with sequences close to the HO cut site compared to *sae2*Δ cells (Figure 3F), although a similar amount of Rad9 can be detected by western blot in all protein extracts (Figure 3E). As the increased Rad9 binding at DSBs in *sae2*Δ cells is due to the hyperactivation of Tel1 (70–72), the diminished Rad9 association with DSBs in *rif2-S6E sae2*Δ cells might be due to a defective Tel1 signalling activity.

### Rif2^S6E^ reduces Tel1 activation by decreasing Tel1 association with DSBs

**Figure 4.**
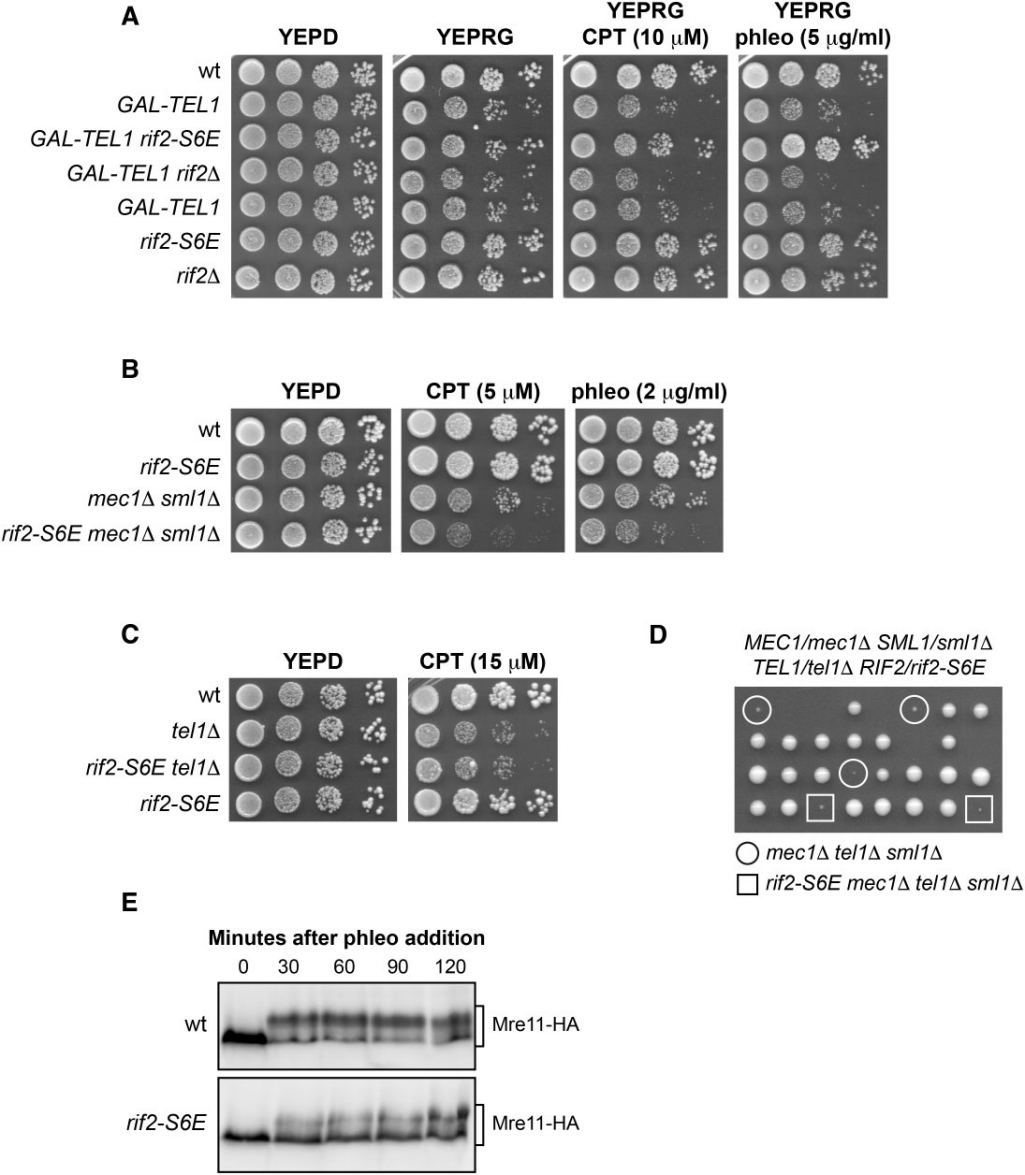
The *rif2-S6E* allele reduces Tel1 activation. (**A**) Exponentially growing cultures were serially diluted (1:10) and each dilution was spotted out onto YEPD plates or onto YEPRG plates with or without CPT or phleomycin. **(B, C)** Exponentially growing cultures were serially diluted (1:10) and each dilution was spotted out onto YEPD plates with or without CPT or phleomycin. (**D**) Meiotic tetrads were dissected on YEPD plates, followed by spore genotyping. (**E**) Phleomycin (100 μg/ml) was added to exponentially growing cells and protein extracts were analyzed by western blot with an anti-HA antibody to detect the Mre11 protein.

Tel1-deficient cells do not exhibit marked sensitivity to DNA damaging agents and show no significant defects in checkpoint activation in response to a single DSB (73). Therefore, to assess the impact of the *rif2-S6E* mutation on Tel1 activation, we employed various approaches. Initially, we leveraged a previous discovery that adding galactose to cells expressing the *TEL1* gene under the galactose inducible *GAL1* promoter (*GAL-TEL1*) triggers a transient checkpoint activation that correlates with an increased MRX association with telomeres (74–76). The cell-cycle arrest induced by this checkpoint activation heightens the sensitivity of *GAL-TEL1* cells to DNA damaging agents by reinforcing the checkpoint-mediated cell-cycle arrest induced by genotoxic exposure. Interestingly, Rif2 was identified as a multicopy suppressor of the DNA damage sensitivity of galactose-induced *GAL-TEL1* cells likely because it counteracts Tel1 activation (75). Therefore, we compared the effects of *rif2*Δ and *rif2-S6E* on the DNA damage sensitivity of *GAL-TEL1* cells in the presence of galactose, which induces Tel1 overexpression. We hypothesized that if the *rif2-S6E* mutation suppresses Tel1 activation more efficiently than wild-type Rif2, it should mimic the effect of *RIF2* overexpression and therefore alleviate the DNA damage sensitivity of galactose-induced *GAL-TEL1* cells. Conversely, *RIF2* deletion is expected to increase this sensitivity by enhancing Tel1 activation. As expected, *GAL-TEL1* cells spotted on galactose containing plates showed a slight growth defect compared to wild-type cells that increases in the presence of CPT or phleomycin (Figure 4A). The *rif2-S6E* allele suppressed the DNA damage sensitivity of *GAL-TEL1* cells, whereas *rif2*Δ slightly increased it (Figure 4A), suggesting that Rif2^S6E^ mutant variant limits Tel1 activation more efficiently than wild-type Rif2.

Next, we took advantage of the finding that the lack of Sae2 suppresses the sensitivity to DNA-damaging agents of *mec1*Δ cells (kept viable by *SML1* deletion) by increasing Tel1 activation (77). This observation prompted us to evaluate the effect of *rif2-S6E* in *mec1*Δ cells. Consistent with a reduced Tel1 activity, when the *rif2-S6E* allele was combined with *mec1*Δ, the double mutant exhibited higher sensitivity to DNA damaging agents than *mec1*Δ alone (Figure 4B). By contrast, *rif2-S6E* did not increase the CPT sensitivity of *tel1*Δ cells (Figure 4C). Furthermore, it failed to suppress the severe growth defect of *mec1*Δ *tel1*Δ (Figure 4D), suggesting that the increased DNA damage sensitivity of *rif2-S6E mec1*Δ is due to a defect in Tel1 activation.

Finally, to confirm an impaired Tel1 signalling activity in *rif2-S6E* cells, we analyzed Mre11 that is known to be specifically phosphorylated by Tel1 and whose phosphorylation results in a decrease of its electrophoretic mobility (78,79). Wild-type cells phosphorylated Mre11 in response to phleomycin treatment, whereas Mre11 phosphorylation was reduced in *rif2-S6E* cells (Figure 4E).

**Figure 5.**
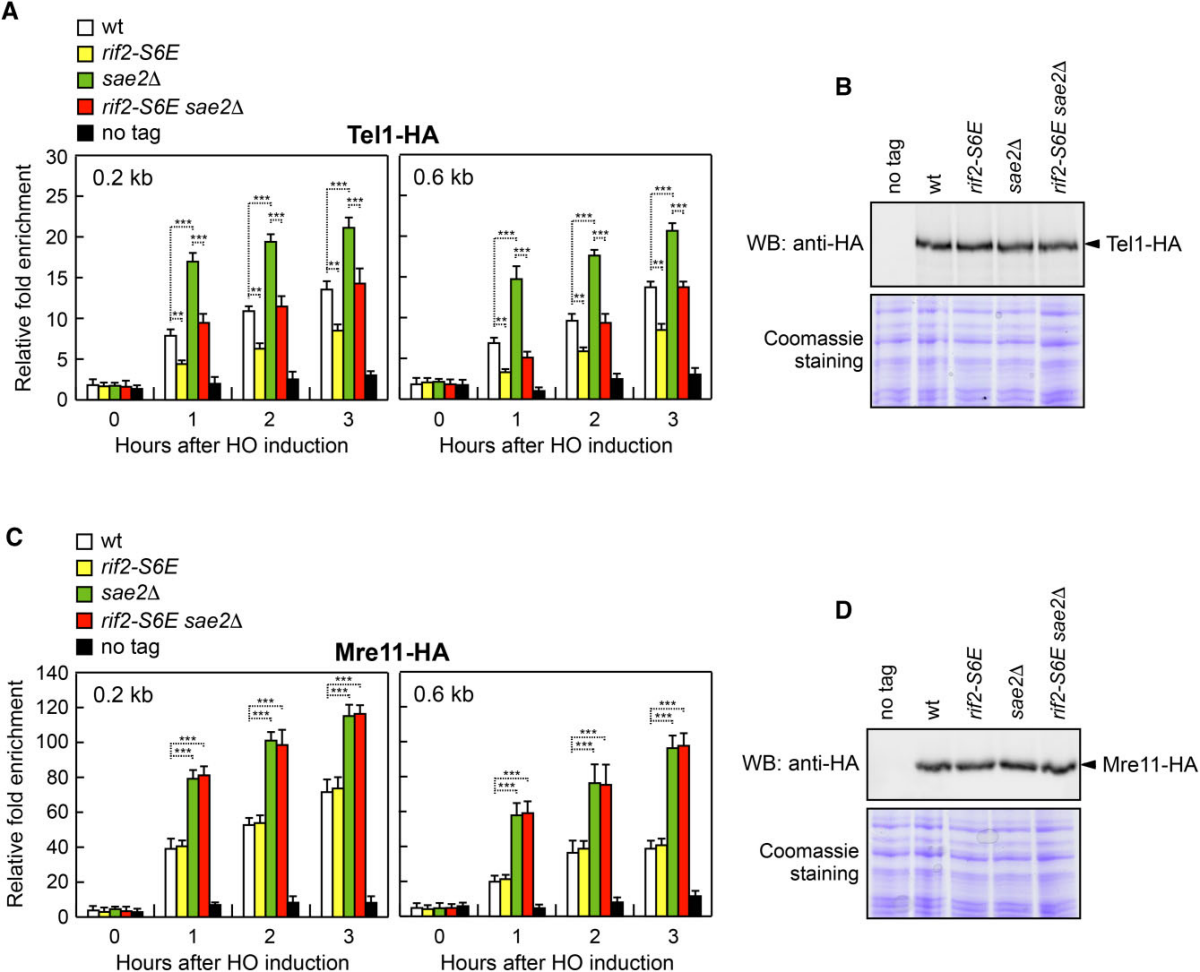
The *rif2-S6E* allele decreases Tel1 but not Mre11 association with the HO-induced DSB. (**A**) Tel1-HA ChIP and qPCR at the indicated distances from the HO-cut site as described in Figure 2F. Plotted values are the mean value of three independent experiments with error bars denoting s.d. ***p< 0.005, **p< 0.01 (Student's *t*-test). (**B**) Western blot with an anti-HA antibody of extracts used for the ChIP analysis shown in (A). (**C**) Mre11-HA ChIP and qPCR at the indicated distances from the HO-cut site as described in Figure 2F. ***p< 0.005 (Student's *t*-test). (**D**) Western blot with an anti-HA antibody of extracts used for the ChIP analysis shown in (C).

The accumulation of MRX and Tel1 is enhanced in *rif2*Δ and *sae2*Δ cells, with *sae2*Δ showing the strongest effect (35,51). To investigate whether the defective Tel1 signalling in *rif2-S6E* cells was due to a decreased MRX and/or Tel1 persistence at DSBs, we measured Mre11 and Tel1 association with sequences close to the HO endonuclease cut site at the *MAT* locus. The presence of the *rif2-S6E* mutation decreased retention of Tel1 at DSBs in both wild-type and *sae2*Δ cells (Figure 5A), although similar amounts of Tel1 were detected in all protein extracts (Figure 5B). By contrast, neither Mre11 binding at the HO-induced DSB nor Mre11 levels was significantly altered in *rif2-S6E* and *rif2-S6E sae2*Δ cells compared to wild-type and *sae2*Δ cells, respectively (Figure 5C and D), indicating that the dampening of Tel1 association with DSBs by Rif2^S6E^ is not due to the inhibition of MRX stable interaction with DNA ends.

### Rif2^S6E^ impairs DSB end-tethering and DSB repair by SSA

**Figure 6.**
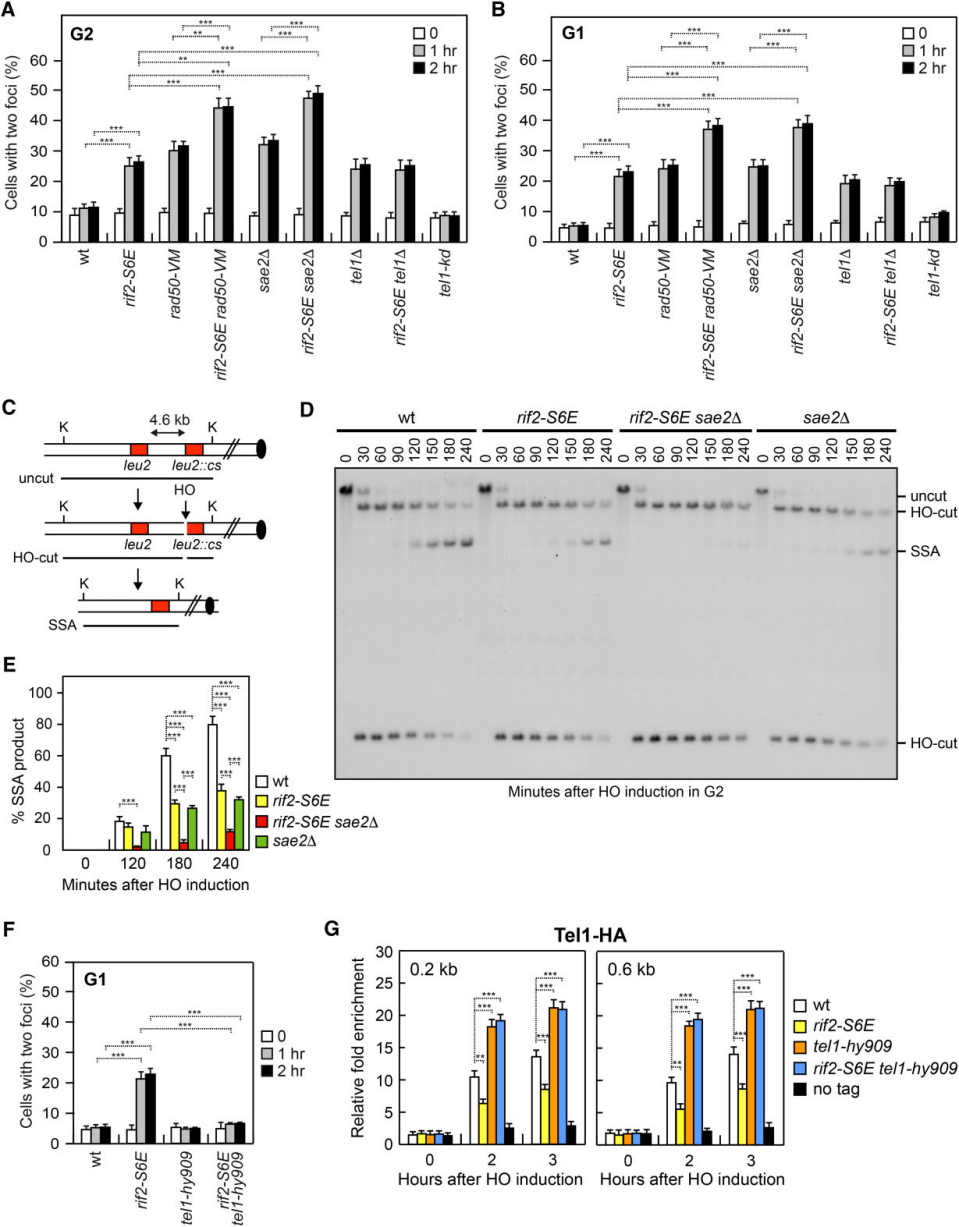
DSB end-tethering and repair by SSA in *rif2-S6E* cells. **(A, B)** DSB end-tethering. Exponentially growing YEPR cell cultures were arrested in G2 with nocodazole (**A**) or in G1 with α-factor (**B**) at time zero and transferred to YEPRG in the presence of nocodazole or α-factor, respectively. 200 cells for each strain were analyzed to determine the percentage of cells showing two LacI-GFP foci. Plotted values are the mean value of three independent experiments with error bars denoting s.d. ***p< 0.005, **p< 0.01 (Student's *t*-test). (**C**) System to detect DSB repair by SSA. HO induction by galactose addition generates a DSB between two homologous *leu2* sequences that are 4.6 kb apart. K, *Kpn*I. (**D**) Exponentially growing YEPR cell cultures were arrested in G2 with nocodazole and transferred to YEPRG. Southern blot analysis of *Kpn*I-digested genomic DNA with a *LEU2* probe revealed a 2.5 and 12 kb DNA fragments (HO-cut) resulting from HO-induced DSB formation. DSB repair by SSA generates an 8 kb fragment (SSA). (**E**) Densitometric analysis of the SSA band signals. Plotted values are the mean value of three independent experiments with error bars denoting s.d. ***p< 0.005 (Student's *t*-test). (**F**) Exponentially growing YEPR cell cultures were arrested in G1 with α-factor at time zero and transferred to YEPRG in the presence of α-factor. 200 cells for each strain were analyzed to determine the percentage of cells showing two LacI-GFP foci. Plotted values are the mean value of three independent experiments with error bars denoting s.d. ***p< 0.005 (Student's *t*-test). (**G**) Tel1-HA ChIP and qPCR at the indicated distances from the HO-cut site as described in Figure 2F. ***p < 0.005, **p < 0.01 (Student's *t*-test).

Our previous finding that *RIF2* deletion increases DSB end-tethering and suppresses the end-tethering defect of *rad50-VM* cells (35) prompted us to examine the effect of the *rif2-S6E* allele on DSB bridging. To detect the ability of cells to keep the DSB ends close to each other, we used a genetic background in which multiple repeats of the LacI repressor binding site are integrated 50 kb upstream and downstream of an HO cleavage site located on chromosome VII in cells constitutively expressing a LacI-GFP fusion protein (11). The level of end-tethering was determined by measuring the generation of one or two LacI-GFP foci, upon expression of HO by galactose addition to cell cultures that were kept blocked in G2 by nocodazole (Figure 6A) or in G1 by α-factor (Figure 6B). Most wild-type cells showed a single LacI-GFP focus both before and after HO induction, indicating their ability to hold the broken DNA ends together (Figure 6A and B). By contrast, *rif2-S6E* cells showed an increase of two LacI-GFP spots compared to wild-type cells (Figure 6A and B), indicating an end-tethering defect.

The maintenance of the DSB ends in close proximity involves MRX, Sae2, and Tel1 (11–14,80). The absence of Tel1 reduces the ability of cells to keep the DSB ends tethered to each other (14) and this defect was exacerbated when *TEL1* was deleted in cells carrying the *rad50-VM* allele (35). Interestingly, opposite to *rif2*Δ that suppresses the end-tethering defect of *sae2*Δ and *rad50-VM* cells (35), the *rif2-S6E* allele exacerbated the end-tethering defect of both *rad50-VM* and *sae2*Δ cells, but not of *tel1*Δ cells (Figure 6A and B), suggesting that Rif2 and Tel1 regulate end-tethering by acting in the same pathway. The role of Tel1 in supporting DSB tethering does not require its kinase activity, as cells expressing a Tel1 kinase-dead allele (*tel1-kd*) did not show an end-tethering defect (Figure 6A and B).

A DSB flanked by direct DNA repeats can be repaired by single-strand annealing (SSA). This mechanism requires that resection reaches the complementary DNA sequences followed by Rad52-dependent annealing of the resulting complementary ssDNA (81). We have previously shown that the lack of Sae2 impairs DSB repair by SSA and the poor SSA efficiency is in part due to the end-tethering defect (80), prompting us to test the ability of *rif2-S6E* cells to repair a DSB by SSA. To measure the efficiency of SSA, we used YMV45 derivative strains carrying tandem repeats of the *LEU2* gene located 4.6 kb apart on chromosome III, with the HO cutting site adjacent to one of the repeats (Figure 6C) (82). HO was induced by galactose addition to exponentially growing cells and galactose was maintained in the medium in order to re-cleave the HO sites that can be rejoined by NHEJ. When DSB repair was monitored by Southern blot analysis with a *LEU2* probe, accumulation of the SSA repair product was reduced in *rif2-S6E* cells compared to wild-type cells (Figure 6D and E), indicating a role for Rif2 in this repair mechanism. The finding that *rif2-S6E* cells did not affect resection of the HO-induced DSB (Figure 3C and D) indicates that the poor SSA efficiency of *rif2-S6E* cells cannot be explained by a resection defect, but it can be due to the low degree of end-tethering. Consistent with this hypothesis, the *rif2-S6E* allele, which increases the end-tethering defect of *sae2*Δ cells (Figure 6A and B), exacerbates the SSA defect of these cells as well (Figure 6D and E). Again, this SSA defect cannot be due to a reduced DSB resection, as *rif2-S6E sae2*Δ cells resect the HO-induced DSB faster than *sae2*Δ cells (Figure 3C and D).

### The end-tethering defect of *rif2-S6E* cells is due to a reduced Tel1 association with DSBs

Tel1 supports end-tethering independently of its kinase activity (Figure 6A and B) and Rif2 counteracts this function by decreasing Tel1 association with DSBs (Figure 5A). If the end-tethering defect of *rif2-S6E* cells is due to a reduced Tel1 association with DSBs, enforcing Tel1 recruitment at DSBs should restore the ability of *rif2-S6E* cells to bridge the DSB ends. To address this question, we used the hypermorphic *tel1-hy909* allele that we identified by searching for *tel1* mutations that compensate for the lack of Mec1 function (83). The *tel1-hy909* allele, which carries the missense mutations A2287V, I2336T, and K2751R, increases Tel1 kinase activity and Tel1 association with DNA DSBs, while leaving Tel1 level within the cell unchanged (53,83). Furthermore, it causes telomere overelongation in an MRX-independent manner (84). When we monitored DSB end-tethering by measuring the generation of one or two LacI-GFP foci upon expression of HO by galactose addition, *tel1-hy909 rif2-S6E* cells showed a decreased percentage of two LacI-GFP spots compared to *rif2-S6E* cells (Figure 6F). Furthermore, the presence of the *tel1-hy909* mutation increased the amount of Tel1 bound at the HO-induced DSB in both wild-type and *rif2-S6E* cells (Figure 6G). These findings indicate that the *tel1-hy909* mutation suppresses the end-tethering defect of *rif2-S6E* cells by increasing Tel1 association with DNA DSBs. This finding, together with the observation that the *rif2-S6E* allele decreased Tel1 association but not MRX binding at DSBs, supports a structural and direct role of Tel1 in DSB end-bridging.

### Rif2^S6E^ limits Tel1–MRX interaction

**Figure 7.**
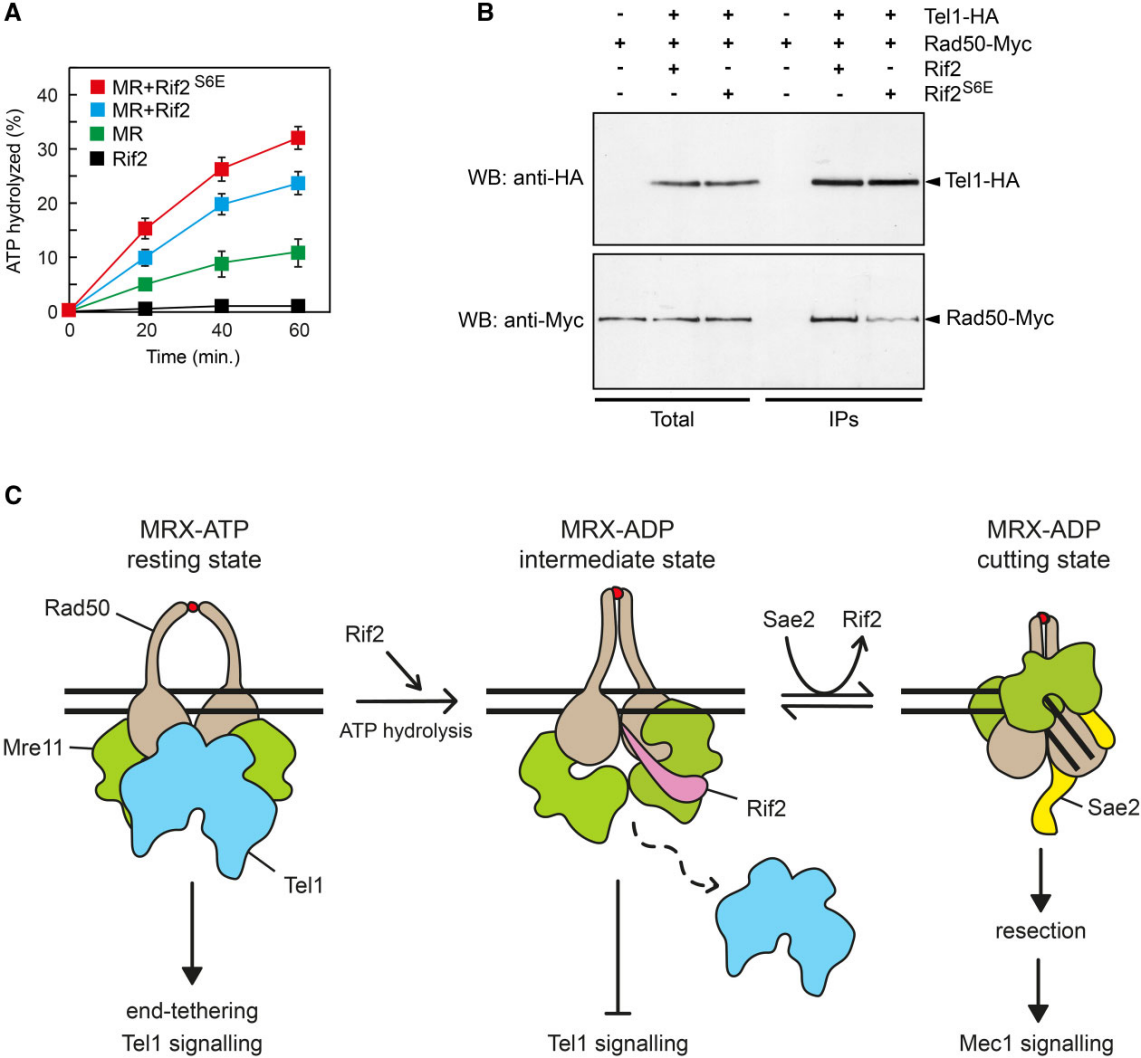
Rif2^S6E^ increases ATP hydrolysis by Rad50 and reduces Tel1-MRX interaction. (**A**) ATPase assays of Mre11-Rad50 subcomplex in the presence of Rif2 or Rif2^S6E^. Plotted values are the mean value of three independent experiments with error bars denoting s.d. (**B**) Protein extracts from exponentially growing cells were analyzed by western blotting with anti-HA and anti-Myc antibodies either directly (Total) or after immunoprecipitation (IPs) of Tel1-HA with an anti-HA antibody. (**C**) Model for Rif2 regulation of Tel1 activity at DNA ends. Tel1 binds the MRX ATP-bound state (resting state) and supports DSB end-tethering. Rif2 binding to the MRX ATP-bound conformation and stimulation of ATP hydrolysis facilitate the transition to an ADP-bound MRX intermediate that is not competent to bind Tel1 and to cleave DNA. Sae2 antagonizes Rif2 binding to Rad50 and stabilizes an ADP-bound MRX conformation (cutting state) that is proficient to initiate DSB resection but is still not able to bind Tel1. The release of Tel1 from Rad50 binding and the generation of ssDNA lead to a transition from a Tel1- to a Mec1-mediated checkpoint signalling. The red dots indicate Zn^2+^ ions.

Because Rif2 is known to stimulate its ATPase activity (35,59), we investigated the effect of the *rif2-S6E* mutation on Rad50 ATPase. The addition of purified Rif2^S6E^ to the Mre11-Rad50 subcomplex increased the ATP hydrolysis activity by Rad50 more efficiently than wild-type Rif2 (Figure 7A), indicating that Rif2^S6E^ possesses an increased ability to stimulate ATPase by Rad50 possibly due to its more robust interaction with Rad50 (Figure 2G).

As Tel1 recruitment at DSBs requires MRX (29–34), we tested whether Rif2^S6E^ binding to Rad50 can disrupt MRX-Tel1 interaction. When Tel1 was immunoprecipitated with an anti-HA antibody, we observed a reduction in Rad50-Myc detected in HA-tagged Tel1 immunoprecipitates from *rif2-S6E* cells compared to wild-type cells (Figure 7B). These findings indicate that the Rif2^S6E^ mutant variant impairs MRX-Tel1 interaction more efficiently than wild-type Rif2, possibly because it possesses an enhanced ability to promote ATP hydrolysis by Rad50 that facilitates the transition to an ADP-bound MRX intermediate incapable to bind Tel1.

## Discussion

Rif2 exerts inhibitory effects on both Mre11 endonuclease activity and activation of the Tel1 kinase. These Rif2 inhibitory properties reside in the MIN motif, a 36 amino acid sequence located at the N-terminus that binds Rad50 ATPase head and stimulates its ATPase activity *in vitro* (50,51,57,59). The available evidence supports a model in which the MIN motif reduces Mre11 nuclease activity by counteracting Sae2 interaction with Rad50 and therefore the stabilization of the Mre11-Rad50 cutting state. However, the mechanisms through which the MIN motif limits Tel1 activation and the *in vivo* implications of this inhibition at DNA DSBs are poorly understood.

To comprehend the outcomes of Rif2 binding to Rad50, we used the multimer algorithm of AlphaFold to provide structural modelling of the interaction interface between the 36 residues of Rif2-MIN with Rad50. The predicted model is consistent with previous mutational data (57) and our engineering of mutations that decrease or increase Rif2-Rad50 interaction strongly supports the accuracy of the predicted interaction interface. Furthermore, the finding that the effect of the *rif2-S6E* allele, which in the model is supposed to strengthen the interaction of Rif2 with Rad50 R125, can be abrogated by changing R125 to K, not only contributes to validating the model but also indicates that the Rif2 inhibitory function depends on a direct interaction between Rad50 and Rif2.

The increased Rif2-Rad50 interaction in *rif2-S6E* cells allowed us to study the *in vivo* consequences of Rif2 binding to Rad50 at DNA DSBs. We found that *rif2-S6E* cells reduce MRX-mediated hairpin cleavage, thus indicating that Rif2 can limit Mre11 endonuclease activity at DSBs not only *in vitro* but also within the cellular environment. The finding that a heightened Rif2 activity at DSBs can limit DNA cleavage by MRX can explain the need for having higher levels of Sae2 than Rif2 at these sites to guarantee that there is an adequate nuclease active Mre11-Rad50 subcomplex to initiate DSB resection.

We also found that the Rif2^S6E^ mutant variant inhibits Tel1 activation by decreasing Tel1 association with DSBs more efficiently than wild-type Rif2. It should be noted that the reduction of Tel1 activity in *rif2-S6E* cells is not enough to shorten telomeres. As Rif2 and its interacting proteins Rif1 and Rap1 have been shown to form a molecular Velcro that covers the telomeric DNA (68), it is possible that the *rif2-S6E* mutation is not capable of further increasing the already severe Rif2-mediated inhibition of Tel1 activity. Conversely, the *rif2-S6E* allele reduces Tel1 association with DSBs to such an extent that Rad9 binding at DSBs is diminished in *sae2*Δ cells, possibly allowing the *rif2-S6E* allele to suppress the *sae2*Δ resection defect.

The low Tel1 association at DSBs in *rif2-S6E* cells reduces the efficiency of DSB end-tethering and the contribution of Tel1 in the maintenance of the DSB ends in close proximity does not require its kinase activity. Given that MRX supports DNA end-tethering (11–14) and Tel1 is known to stabilize MRX persistence with DSBs (35,36), the end-tethering defect resulting from diminished Tel1 association with DSBs in *rif2-S6E* cells might be attributed to lower Mre11 retention at DSBs. However, the finding that the *rif2-S6E* mutation reduces Tel1 association with DSBs without diminishing MRX association at these sites indicates that the decreased end-tethering in *rif2-S6E* cells is not caused by reduced MRX persistence at DSBs. This observation, together with the finding that increasing Tel1 enrichment at DSBs by expressing the Tel1^hy909^ mutant variant is enough to suppress the end-tethering defect of *rif2-S6E* cells, supports a direct and structural role of Tel1 in bridging DSB ends.

The finding that the Rif2^S6E^ mutant variant decreases Tel1 binding at DSBs, whereas MRX association with DSBs remains unaffected, also indicates that Rif2 can negatively regulate Tel1 recruitment at DSBs independently of the control of MRX persistence at the DNA ends. Consistent with such a role, Rif2^S6E^ reduces the physical association between Tel1 and MRX, indicating that Rif2 limits Tel1 activation by antagonizing MRX-Tel1 interaction. Biochemical studies have shown that Rif2 stimulates the ATPase activity by Rad50 (35,59). As Rif2^S6E^ stimulates Rad50 ATPase more efficiently than wild-type Rif2, these data suggest that Rif2 binding to Rad50 and stimulation of its hydrolysis induce a conformational state of Mre11-Rad50 that is not competent for Tel1 binding and therefore for stimulation of its kinase activity.

These data support a model where the ATP-bound form of MRX (resting state) is effective at interacting with Tel1 and tethering the DSB ends (Figure 7C). In this model, the association of Rif2 with Rad50 stimulates the ATP hydrolysis by Rad50, facilitating the transition to an ADP-bound MRX intermediate state, which is incapable to bind Tel1 and to perform its endonuclease function. The high amount of Sae2 bound at DSBs compared to Rif2 displaces Rif2 from its association with Rad50. Sae2 binding to Rad50 stabilizes MRX in a nucleolytically active ADP-bound conformation (cutting state), capable of initiating DSB resection yet still unable to bind Tel1. Therefore, the ATPase activity of Rad50 on one hand allows MRX-mediated nucleolytic degradation of DSBs, and on the other hand, releases Tel1 from Rad50 binding, thus explaining a previous observation that the generation of ssDNA at the DSB ends leads to a transition from Tel1- to Mec1-mediated checkpoint signalling (73). Conversely, at normal-length telomeres, the presence of a Rif2-mediated higher-order structure locks MRX in an ADP-bound state that neither binds Tel1 nor exhibits MRX nuclease activity, preventing unwanted telomere elongation and checkpoint activation. Considering that mammalian TRF2 suppresses ATM signalling (85), it would be intriguing to explore whether this suppression is mediated through the TRF2 iDDR motif.

## Supplementary Material

gkad1246_Supplemental_File

## Data Availability

All relevant data are included in the manuscript and the Supplementary Data file. Any other data are available from the authors on request.
