# Supplementary material for: Rif2 interaction with Rad50 counteracts Tel1 functions in checkpoint signalling and DNA tethering by releasing Tel1 from MRX binding
Source: Nucleic Acids Res. 2024 Jan 5;52(5):2355–71. doi: 10.1093/nar/gkad1246 (PMC10954470; doi:10.1093/nar/gkad1246)
Supplement: gkad1246_Supplemental_File [file gkad1246_supplemental_file.pdf]

SUPPLEMENTARY INFORMATION

Table S1. ***Saccharomyces cerevisiae*** **strains used in this study.**

**Strain**	**Relevant Genotype**	**Source**
W303	*MAT*a/α *ade2-1 can1-100 his3-11,15 leu2-3,112 trp1-1 ura3-1 rad5-535*	
YLL 1134.2	W303 *MAT*a *rif2*Δ*::KANMX*	(1)
YLL 4501.2	W303 *MAT*a *rif2-S6E::LEU2*	This study
YLL 4503.7	W303 *MAT*a *rif2-F8E::LEU2*	This study
YLL 4504.2	W303 *MAT*a *rif2-V18E::LEU2*	This study
YLL 4505.8	W303 *MAT*a *rif2-I23E::LEU2*	This study
DMP 5781/1B	W303 *MAT*a *rad50-V1269M::KANMX*	(1)
DMP 7823/1C	W303 *MAT*a *rif2-S6E::LEU2 rad50-V1269M::KANMX*	This study
DMP 7824/6C	W303 *MAT*a *rif2-F8E::LEU2 rad50-V1269M::KANMX*	This study
DMP 7825/1A	W303 *MAT*a *rif2-V18E::LEU2 rad50-V1269M::KANMX*	This study
DMP 7826/1A	W303 *MAT*a *rif2-I23E::LEU2 rad50-V1269M::KANMX*	This study
YLL 1133	W303 *ura3::TEL1::URA3 tel1::GAL1-TEL1::LEU2*	(2)
DMP 7827/2D	W303 *GAL1-TEL1::LEU2 rif2-S6E::LEU2*	This study
DMP 7828/1D	W303 *GAL1-TEL1::LEU2 rif2*Δ*::KANMX*	This study
YLL 490.4	W303 *mec1*Δ*::HIS3 sml1*Δ*::KANMX*	(3)
DMP 7829/4A	W303 *mec1*Δ*::HIS3 sml1*Δ*::KANMX rif2-S6E::LEU2*	This study
DMP 3335/2A	W303 *tel1*Δ*::HIS3*	(4)
DMP 7830/2A	W303 *tel1*Δ*::HIS3 rif2-S6E::LEU2*	This study
YLL 4720	W303 *rad50-R125K-VM::KANMX*	This study
DMP 7871	W303 *rad50-R125K-VM::KANMX rif2-S6E::LEU2*	This study
HS21	MATα *ade5-1 his7-2 ura3Δ trp1-289 leu2-3,112::p305L3 LEU2 lys2::AluIR*	(5)
YLL 4694.1	HS21 *MAT*a *rif2*Δ*::KANMX*	This study
YLL 4348.1	HS21 MATα *mre11-H15N::LEU2*	(6)
YLL 4695.2	HS21 *MAT*a *rif2-S6E::HPHMX*	This study
JKM139	*MAT*a *hml*Δ*::ADE1, hmr*Δ*::ADE1, ade1-100, lys5, leu2-3,112, trp1::hisG ura3-52, ho, ade3::GAL-HO site*	(7)
YLL 4693	JKM139 *MAT*a *rif2-S6E::LEU2*	This study
YLL 1523.3	JKM139 *MAT*a *sae2*Δ*::KANMX*	(8)
DMP 7831/2B	JKM139 *MAT*a *rif2-S6E::LEU2 sae2*Δ*::KANMX*	This study
YLL 3611.1	JKM139 *MATa RIF2-18MYC::TRP1*	(1)
YLL 4696.2	JKM139 *MAT*a *rif2-S6E-18MYC:: TRP1::LEU2*	This study
YLL 3421.2	JKM139 *MAT*a *RAD9-3HA::TRP1*	(9)
DMP 6911/4B	JKM139 *MAT*a *RAD9-3HA::TRP1 sae2*Δ*::KANMX*	(10)
DMP 7832/2A	JKM139 *MAT*a *RAD9-3HA::TRP1 rif2-S6E::LEU2*	This study
DMP 7832/12B	JKM139 *MAT*a *RAD9-3HA::TRP1 rif2-S6E::LEU2 sae2*Δ*::KANMX*	This study
YLL 3188.3	JKM139 *MAT*a *MRE11-3HA::URA3*	(11)
DMP 6867/1B	JKM139 *MAT*a *MRE11-3HA::URA3 sae2*Δ*::KANMX*	This study
DMP 7833/7C	JKM139 *MAT*a *MRE11-3HA::URA3 rif2-S6E::LEU2*	This study
DMP 7833/4B	JKM139 *MAT*a *MRE11-3HA::URA3 rif2-S6E::LEU2 sae2*Δ*::KANMX*	This study
YLL 3222.6	JKM139 *MAT*a *TEL1-3HA::NATMX*	(11)

DMP 6435/1A	JKM139 *MAT*a *TEL1-3HA::NATMX sae2Δ::KANMX*	(6)
DMP 7834/5C	JKM139 *MAT*a *TEL1-3HA::NATMX rif2-S6E::LEU2*	This study
DMP 7834/2B	JKM139 *MAT*a *TEL1-3HA::NATMX rif2-S6E::LEU2 sae2Δ::KANMX*	This study
DMP 7837/1B	JKM139 *MATa RAD50-3HA::URA3 RIF2-18MYC::TRP1*	This study
DMP 7838/5B	JKM139 *MATa RAD50-3HA::URA3 rif2-S6E-18MYC::TRP1::LEU2*	This study
DMP 6905/1C	JKM139 *MATa RAD50-18MYC::URA3 TEL1-3HA::NATMX*	(12)
DMP 7839/2A	JKM139 *MATa RAD50-18MYC::URA3 TEL1-3HA::NATMX rif2-S6E::LEU2*	This study
YLL 3786.1	JKM139 *MATa RAD50-18MYC::URA3*	(12)
YLL 4716	JKM139 *NAT::tel1-hy909-HA::KANMX*	This study
YLL 4717	JKM139 *NAT::tel1-hy909-HA::KANMX rif2-S6E::LEU2*	This study
YJK40.6	*MATΔ hmlΔ hmrΔ can1 lys5 ade2 leu2 trp1 ura3 his3 ade3:: GAL-HO VII::TRP1-HO LacI-GFP::URA3 LacO::LYS5 LacO::KanR*	(13)
YLL 1709.11	YJK40.6 *sae2Δ::NATMX*	(14)
YLL 4697.3	YJK40.6 *rif2-S6E::HPHMX*	This study
YLL 4698.1	YJK40.6 *sae2Δ::NATMX rif2-S6E::LEU2*	This study
YLL 3641.6	YJK40.6 *rad50-V1269M::HPHMX*	(1)
YLL 4699.1	YJK40.6 *rad50-V1269M::HPHMX rif2-S6E::LEU2*	This study
YLL 3617.2	YJK40.6 *tel1Δ::NATMX*	(1)
YLL 4700.2	YJK40.6 *tel1Δ::NATMX rif2-S6E::LEU2*	This study
YLL 4589.4	YJK40.6 *tel1-kd::LEU2*	(15)
YLL 4706	YJK40.6 *tel1-hy909::LEU2*	This study
YLL 4707	YJK40.6 *tel1-hy909::LEU2 rif2-S6E::HPHMX*	This study
YMV45	*ho hml::ADE1 mata::hisG hmr::ADE1 leu2::leu2(Asp718-SalI)- URA3-pBR332-MATa ade3::GAL::HO ade1 lys5 ura3-52 trp1::hisG*	(16)
YLL 1621.9	YMV45 *sae2Δ::KANMX*	(8)
YLL 4701.2	YMV45 *rif2-S6E::LEU2*	This study
YLL 4702.3	YMV45 *sae2Δ::HPHMX rif2-S6E::LEU2*	This study
YAB125	PJ69-4A *MATa trp1-901 leu2-3,112 ura3-52 his3-200 gal4 gal80 LYS2::GAL1-HIS3 GAL2-ADE2 met2::GAL7-lacZ*	(17)

REFERENCES

1. Cassani,C., Gobbini,E., Wang,W., Niu,H., Clerici,M., Sung,P. and Longhese,M.P. (2016) Tel1 and Rif2 regulate MRX functions in end-tethering and repair of DNA double-strand breaks. *PLoS Biol.*, **14**, e1002387.

2. Viscardi,V., Baroni,E., Romano,M., Lucchini,G. and Longhese,M.P. (2003) Sudden telomere lengthening triggers a Rad53-dependent checkpoint in *Saccharomyces cerevisiae*. *Mol. Biol. Cell*, **14**, 3126–3143.

3. Longhese,M.P., Paciotti,V., Neecke,H. and Lucchini,G. (2000) Checkpoint proteins influence telomeric silencing and length maintenance in budding yeast. *Genetics*, **155**, 1577–1591.

4. Casari,E., Gobbini,E., Gnugnoli,M., Mangiagalli,M., Clerici,M. and Longhese,M.P. (2021) Dpb4 promotes resection of DNA double-strand breaks and checkpoint activation by acting in two different protein complexes. *Nat. Commun.*, **12**, 4750.

5. Lobachev,K.S., Gordenin,D.A. and Resnick,M.A. (2002) The Mre11 complex is required for repair of hairpin-capped double-strand breaks and prevention of chromosome rearrangements. *Cell*, **108**, 183–193.

6. Marsella,A., Gobbini,E., Cassani,C., Tisi,R., Cannavo,E., Reginato,G., Cejka,P. and Longhese,M.P. (2021) Sae2 and Rif2 regulate MRX endonuclease activity at DNA double-strand breaks in opposite manners. *Cell Rep.*, **34**, 108906.

7. Lee,S.E., Moore,J.K., Holmes,A., Umezu,K., Kolodner,R.D. and Haber,J.E. (1998) *Saccharomyces* Ku70, Mre11/Rad50, and RPA proteins regulate adaptation to G2/M arrest after DNA damage. *Cell*, **94**, 399–409.

8. Gobbini,E., Villa,M., Gnugnoli,M., Menin,L., Clerici,M. and Longhese,M.P. (2015) Sae2 function at DNA double-strand breaks is bypassed by dampening Tel1 or Rad53 activity. *PLoS Genet.*, **11**, 1005685.

9. Clerici,M., Trovesi,C., Galbiati,A., Lucchini,G. and Longhese,M.P. (2014) Mec1/ATR regulates the generation of single-stranded DNA that attenuates Tel1/ATM signaling at DNA ends. *EMBO J.*, **33**, 198–216.

10. Colombo,C.V., Menin,L., Ranieri,R., Bonetti,D., Clerici,M. and Longhese,M.P. (2019) Uncoupling Sae2 functions in downregulation of Tel1 and Rad53 signaling activities. *Genetics*, **211**, 515–530.

11. Cassani,C., Gobbini,E., Vertemara,J., Wang,W., Marsella,A., Sung,P., Tisi,R., Zampella,G. and Longhese,M.P. (2018) Structurally distinct Mre11 domains mediate MRX functions in resection, end-tethering and DNA damage resistance. *Nucleic Acids Res.*, **46**, 2990–3008.

12. Cassani,C., Vertemara,J., Bassani,M., Marsella,A., Tisi,R., Zampella,G. and Longhese,M.P. (2019) The ATP-bound conformation of the Mre11-Rad50 complex is essential for Tel1/ATM activation. *Nucleic Acids Res.*, **47**, 3550–3567.

13. Kaye,J.A., Melo,J.A., Cheung,S.K., Vaze,M.B., Haber,J.E. and Toczyski,D.P. (2004) DNA breaks promote genomic instability by impeding proper chromosome segregation. *Curr. Biol.*, **14**, 2096–2106.

14. Clerici,M., Mantiero,D., Lucchini,G. and Longhese,M.P. (2005) The Sae2 protein promotes resection and bridging of double strand break ends. *J. Biol. Chem.*, **280**, 38631–38638.

15. Rinaldi,C., Pizzul,P., Casari,E., Mangiagalli,M., Tisi,R. and Longhese,M.P. (2023) The Ku complex promotes DNA end-bridging and this function is antagonized by Tel1/ATM kinase. *Nucleic Acids Research*, **51**, 1783–1802.

16. Vaze,M.B., Pellicioli,A., Lee,S.E., Ira,G., Liberi,G., Arbel-Eden,A., Foiani,M. and Haber,J.E. (2002) Recovery from checkpoint-mediated arrest after repair of a double-strand break requires Srs2 helicase. *Mol Cell*, **10**, 373–385.

17. Khayat,F., Cannavo,E., Alshmery,M., Foster, W.R., Chahwan,C., Maddalena,M., Smith,C., Oliver,A. W., Watson, A. T., Carr, A. M., Cejka, P., and Bianchi, A. (2021). Inhibition of MRN activity by a telomere protein motif. *Nat. Comm.*, **12**(1), 3856.

Table S2. Oligonucleotides used for gene tagging and disruptions.

Name	Sequence (5'-3')
PRP330	TGAATAAAGAATGATGATCGCTGGCGTTTAACATCTAGCATATATCTGCAATAATTTATCACTCATCGATGAATTCGAGCTCG
PRP331	TGTAAGCCATTAGGTGTTTGTATGTGAGATGGTGACTGGTGAAGAAAAGTGTATCTAAAGCGTACGCTGCAGGTCGAC
PRP368	CATAGTGCTTATAACTTTAAGAAAAACCAGCGTCTTCCACTTAAGTTAACTCGAAAAGTACATGATAGATCCGGTTCTGCTGCTAG
PRP369	ACTTGAACTTTTCCAAAGGAGTTGCCATCTCTTTGTATTGTTCGAACTCTTTCAAAAGACCTTGGTAATCCTCGAGGCCAGAAGAC
PRP378	GTGGTTAGTATATAGAGACAATGGAGCATGTAGATTCCGATTTTGCGTACGCTGCAGGTCGAC
PRP379	TCTTTCAAAAGACCTTGGTAATTTATCTATCATGTACTTTTCGAGATCGATGAATTCGAGCTCG
PRP614	TTAGCAGTAGTACCTGAATTAATGGTCAAAGATGGTTCCAAAAGTTTATTGGATGCCGTTTCAGTACATTCGTACGCTGCAGGTCGAC
PRP615	CGTTGTTACTTATGAACTTGCTGACGTTATCGAAATTTGTAATTTCATGTTCTTCTTCAAATAAGTGTTCATCGATGAATTCGAGCTCG
PRP730	ATGATATTACGGACAATGATATATACAACACTATTTCTGAGGTTAGATCCTCGAGGCCAGAAGAC
PRP731	TTAATCGTCCCTTTCTATCAATTATGAGTTTATATATTTTTATAATTCCTCGAGGCCAGAAGAC
PRP890	CTTCAAAGTCAAGAGAGATGATAGGCAAAAATCACAAATTGAGTGGGTCGATATTAACAGAGTCACTTATTCCGGTTCTGCTGCTAG
PRP891	CGACGAAGTATTTCAGCACCTCTAATTAATCAATCAAAGTGTATCCCTTCGTAGATATTATGGGGTCTTTCCTCGAGGCCAGAAGAC
PRP1167	CGAACGTGGTTAGTATATAGAGACAATGGAGCATGTAGATTCCGATTTTGGCTGCTAACGTCTTGCC
PRP1168	GTCAGTCTGTTTCTGATCTTATCCAACAATTCATCAAAAGTTCTACTCTGCCTCGAGGCCAGAAGAC
PRP 2758	AGACAATGGAGCATGTAGATGAGGATTTTGCACGTATAAGGAG
PRP2759	CTCCTTATAGGTGCAAAATCCTCATCTACATGCTCCATTGTCT
PRP 2764	AGGTTGTTGACAGTGACAAGGAAGTGAAAGCAATAAGCGATGA
PRP 2765	TCATCGCTTATTGCTTTCACTTCCTTGTCACTGTCAACAACCT
PRP 3004	CTATAAATAATAGTGGTGACAAAAGTACTTTGTCCACCCGGTC
PRP 3005	GACCGGGTGGACAAAGTACTTTTGTCACCACTATTATTTATAG

Table S3. Oligonucleotides used for qPCR.

Name	Sequence (5'-3')
ARO fwr	TGAGTCGTTACAAGGTGATGCC
ARO rev	ACCTACAGGAGGACCCGAAA
DSB 0.2 fwr	TCAGACTCAAGCAAACAATCAA
DSB 0.2 rev	CCCGTATAGCCAATTCGTTC
DSB 0.6 fwr	CACCCAAGAAGGCGAATAAG
DSB 0.6 rev	CATGCGGTTCACATGACTTT

Figure S1

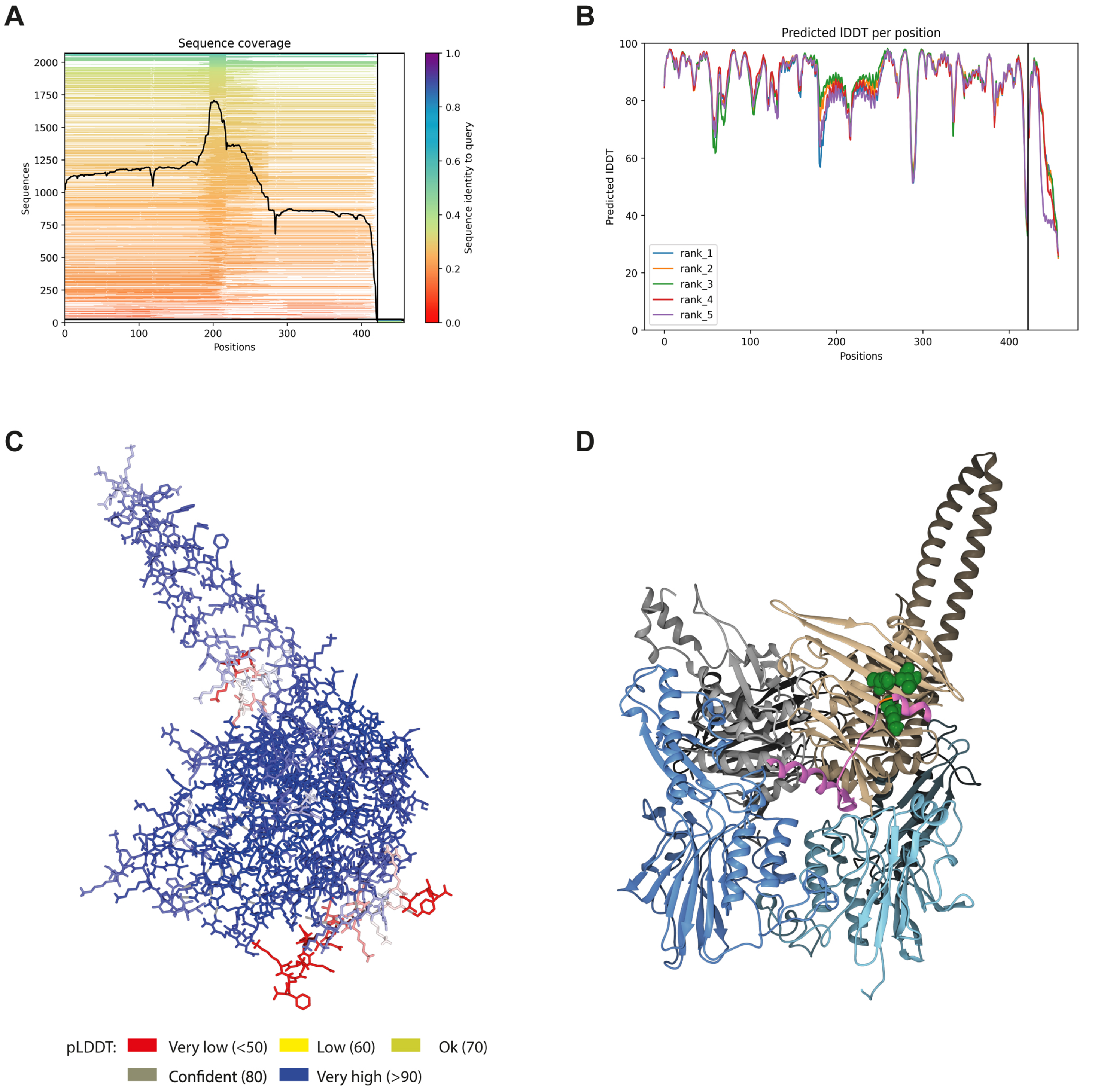
A
Sequence coverage
Sequences
Positions
Sequence identity to query
B
Predicted lDDT per position
Predicted lDDT
Positions
rank_1
rank_2
rank_3
rank_4
rank_5
C
D
pLDDT:
Very low (<50)
Low (60)
Ok (70)
Confident (80)
Very high (>90)

SUPPLEMENTARY FIGURE LEGENDS

Figure S1. Quality assessment of the AlphaFold-multimer generated Rad50-Rif2 N-terminal complex. (**A**) Coverage from the multiple sequence alignment. Rad50 sequence is on the left section, Rif2 on the right. (**B**) LDDT parameter for the 5 top-ranked models. (**C**) LDDT-colored representation of the model. The most C-terminal region of Rif2 fragment is predicted at very low confidence. (**D**) The model of Rad50-Rif2 N-terminal complex was superimposed to one of the two Rad50 in a heterotetrameric Mre11-Rad50 complex, and is represented as a tan cartoon for Rad50 and a pink cartoon for Rif2. The amino acid from Rad50 previously known to be involved in this interaction are represented as green spheres. The other Rad50 subunit is represented as a grey cartoon, while the two Mre11 subunits are in blue and light blue.
